# The role of CBL family ubiquitin ligases in cancer progression and therapeutic strategies

**DOI:** 10.3389/fphar.2024.1432545

**Published:** 2024-07-26

**Authors:** Jiaqi Ren, Linlin Lv, Xufeng Tao, Xiaohan Zhai, Xuyang Chen, Hao Yu, Xinya Zhao, Xin Kong, Zhan Yu, Deshi Dong, Jing Liu

**Affiliations:** ^1^ Department of Pharmacy, The First Affiliated Hospital of Dalian Medical University, Dalian, China; ^2^ School of Pharmacy, Dalian Medical University, Dalian, China; ^3^ Stem Cell Clinical Research Center, National Joint Engineering Laboratory, The First Affiliated Hospital of Dalian Medical University, Dalian, China

**Keywords:** CBL, cancer, E3 ubiquitin ligase, targeted therapy, immunotherapy

## Abstract

The CBL (Casitas B-lineage lymphoma) family, as a class of ubiquitin ligases, can regulate signal transduction and activate receptor tyrosine kinases through various tyrosine kinase-dependent pathways. There are three members of the family: c-CBL, CBL-b, and CBL-c. Numerous studies have demonstrated the important role of CBL in various cellular pathways, particularly those involved in the occurrence and progression of cancer, hematopoietic development, and regulation of T cell receptors. Therefore, the purpose of this review is to comprehensively summarize the function and regulatory role of CBL family proteins in different human tumors, as well as the progress of drug research targeting CBL family, so as to provide a broader clinical measurement strategy for the treatment of tumors.

## 1 Introduction

Some diseases, including cancer, are controlled by E3 ubiquitin ligase, which regulates cell signaling and protein stabilization (Li W. et al., 2022). The CBL protein family consists of the members that function as E3 ubiquitin ligases. Through ubiquitination active receptor tyrosine kinases (RTKs), it negatively regulates the signaling, causing it to degrade in lysosomes. In addition, CBL-mediated ubiquitination may alter cell localization without degrading signaling proteins. In addition, the CBL protein also functions as a receptor protein that enlists signaling molecules into the active RTKs (Liyasova et al., 2015). It plays a primary role in the onset and progression of cancer, hematopoietic development and the regulation of T cell receptors.

c-CBL is a member of the CBL protein family and is comprised of three homologous genes. c-CBL is a universally expressed mammalian gene that plays an essential role in fundamental cellular processes, including cell survival. New research indicates that CBL-b not only regulates the signaling of receptor protein tyrosine kinase (RTK) but also has an impact on innate immune pathways. It has been found to modulate the signaling cascades initiated by various innate immune receptors, such as toll-like receptors (TLRs) and cytokine receptors, which plays a crucial role in initiating the immune responses against pathogens. In addition, CBL-c mutation may lead to the development of epithelial malignant tumors, and CBL-c is closely related to the development of tumors. This review comprehensively and specifically summarizes how CBL affects the occurrence and development of glioma, lung cancer, liver cancer, pancreatic cancer, gastric cancer, colorectal cancer, kidney cancer, prostate cancer, thyroid cancer, ovarian cancer, and breast cancer, and lists some anti-tumor drugs related to CBL. The aim is to find new tumor treatment methods and provide new therapeutic means for clinical practice.

## 2 Structure

**FIGURE 1:**
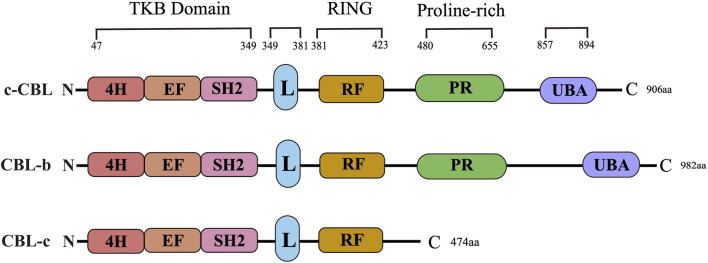
CBL family structure. The primary structure and domain organization of human Casitas b-lineage lymphoma family proteins. c-CBL, CBL-b and CBL-c proteins contain highly conserved tyrosine kinase-binding (TKB), linker (L), RING finger (RF) and proline-rich (PR) domains. 4H: Four-helix bundle; EF: EF hand, SH2: Src-homology 2; UBA: Ubiquitin-associated domain.

CBL proteins are part of a highly conserved family of ubiquitin ligases (E3s) known for their characteristic ring finger domain. The CBL protein family is comprised of three well-known members: c-CBL, CBL-b, and CBL-c. The structural composition of the CBL protein includes an N-terminal tyrosine kinase binding (TKB) domain, a linker region, a ring domain, a proline enrichment region (PRR), a C-terminal phosphorylation site, and a ubiquitin-associated domain. The CBL family has a highly conserved structure at the N-terminal, consisting of a tyrosine kinase binding (TKB) domain, an α-helical linking domain, and a catalytic ring finger domain (Figure 1). However, compared with c-CBL and CBL-b, CBL-c lacks a region including proline-rich (PR) regions, followed by amino acid residues with several serine and tyrosine phosphorylation sites, and a ubiquitin binding domain. The TKB domain is composed of three essential components: a 4-helix bundle (4H), a calcium-binding domain exhibiting EF-hand folding, and a mutated Src Homology region 2 (SH2) domain. These three elements comprise a distinctive phosphotyrosine-binding (PTB) module. Both the conserved short-ligand subdomain and the proper functioning of the link-TKB interface are essential for the E3 ligase activity and transformation potential of c-CBL.

Recent studies have demonstrated that three N-terminal tyrosine residues (Y106 and Y133 within the TKB domain, as well as Y363 in the ligand region) play a vital role in the E3 ubiquitin ligase activity of CBL-b. Research has revealed that the phosphorylation of CBL-b at Y363 plays a critical role in regulating its E3 ubiquitin ligase activity. This phosphorylation event can lead to the unmasking of the RF domain within the TKB domain, thereby enhancing the catalytic effect of CBL-b. The RF domains, which are highly conserved, possess an intrinsic E3 ligase activity and are capable of recruiting E2 ubiquitin-conjugating enzymes to facilitate the transfer of ubiquitin to the target substrate. The proper structural integrity of the RF domain is essential for the functioning of the CBL protein as an E3 ubiquitin ligase (Tang et al., 2019). Likewise, CBL-b exhibits the comparable phosphorylation sites to c-CBL, such as Y655 and Y709, all of which bear sequence homology to Y700 and Y774 of c-CBL, respectively. As a consequence, both c-CBL and CBL-b are selective targets for a similar spectrum of protein tyrosine kinases (PTKs) and have the capacity to engage with the proteins containing SH2 domains.

A major distinction between c-CBL and CBL-b is the presence of a specific tyrosine residue, namely, Y731, which is exclusively found in c-CBL. This residue has the ability to bind to the SH2 domain of the p85 subunit of PI3K, thereby exerting a regulatory effect on the function of PI3K. Also, CBL-b lacks this particular tyrosine residue in its sequence. The ubiquitin-associated (UBA) domain, which is a conserved domain present in the C-termini of both c-CBL and CBL-b, plays a crucial role. However, it is noteworthy that CBL-c does not possess this UBA domain. The UBA domain allows for the interaction between c-CBL and CBL-b, facilitating their homodimerization and heterodimerization. This interaction is enabled by the UBA domains of c-CBL and CBL-b, both of which have the capability to bind to and interact with each other. The role and significance of UBA domains in the E3 ubiquitin ligase activity of c-CBL and CBL-b are not yet fully understood. It has been demonstrated that the UBA domains are dispensable for the E3 ubiquitin ligase function of both c-CBL and CBL-b. However, further research is required to elucidate the exact function and importance of UBA domains in the overall ubiquitination processes mediated by c-CBL and CBL-b. Additionally, it has been found out that the UBA domain of CBL-b exhibits a higher affinity to free poly-ubiquitin chains compared to monoubiquitin. This distinctive binding preference allows the UBA domain of CBL-b to limit various ubiquitin-mediated processes, including the degradation of ubiquitinated proteins. This disparity in ubiquitin-binding capacity further highlights a contrast between c-CBL and CBL-b in terms of their functional properties and interactions with ubiquitin-modified substrates.

## 3 Critical roles of CBL family proteins in various cancer

**FIGURE 2:**
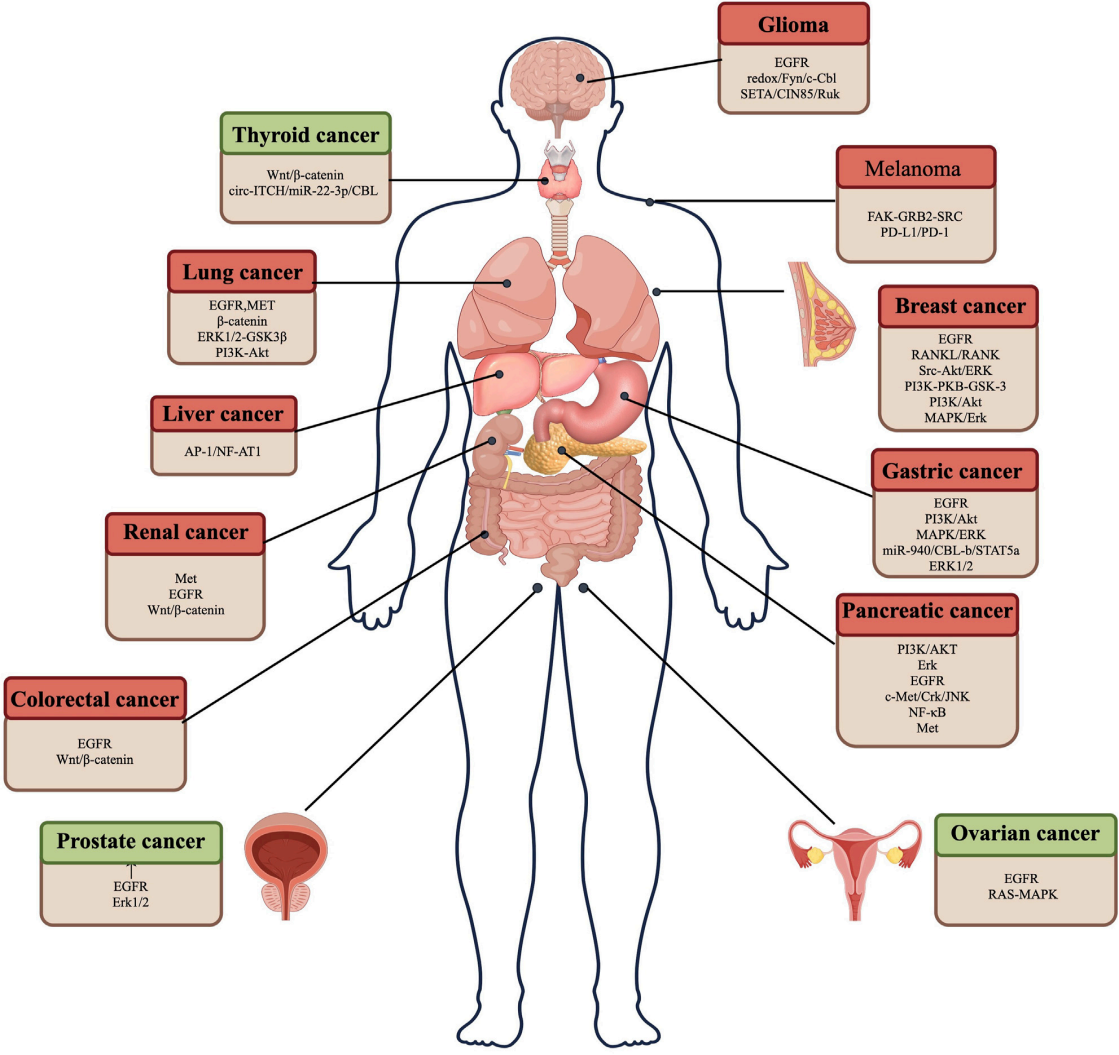
CBL plays a role in promoting and suppressing cancer by regulating different pathways in various tumor cells. Red represents the role of CBL in promoting cancer, and green represents the role of CBL in suppressing cancer.

**FIGURE 3:**
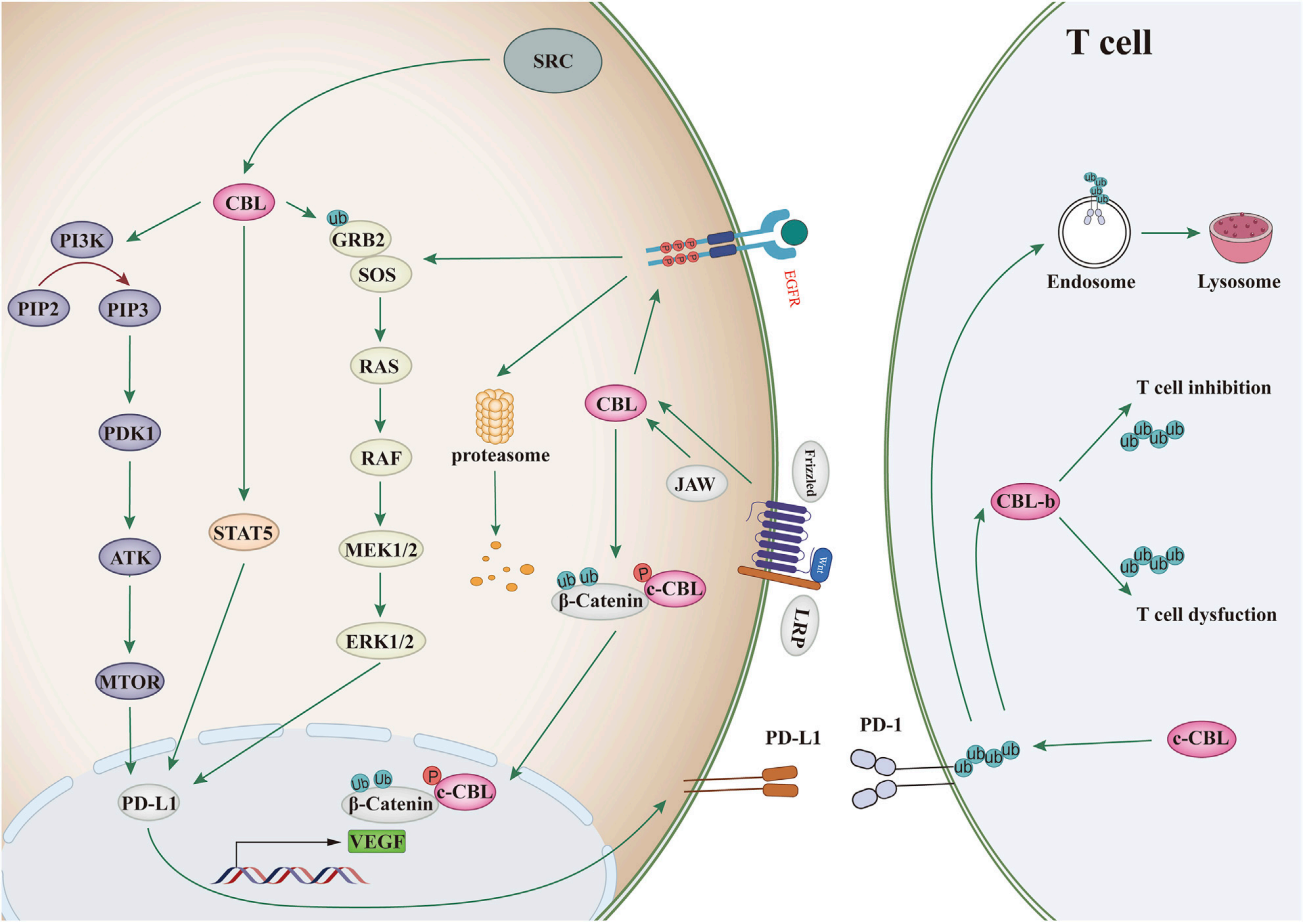
CBL affects the signaling pathway. Binding of ligand (EGF) to EGF receptor leads to aggregation of multiple receptors. This allows for docking of proteins, leading to activation of the Ras/Raf/Erk, PI3K/Akt, and JAK pathways. c-CBL regulates angiogenesis through Wnt signaling.

CBL family proteins can regulate signal transduction through various tyrosine kinase-dependent pathways and activate receptor tyrosine kinases. Moreover, CBLC family proteins play different regulatory roles in different human tumors (Figures 2, 3 and Table 1).

**TABLE 1:** CBL is associated with pathways in different cancers.

Classify	Types	Cell model	Signaling pathway	References
Glioma	c-CBL CBL-b	U-87MG, LN229, T98G U251, A172 SHG-44, SU2	EGFR redox/Fyn/c-Cbl SETA/CIN85/Ruk	Zhang et al. (2013a) Stevens et al. (2014) Schmidt et al. (2003)
Lung cancer	c-CBL CBL-b CBL-c	PC9, H1975, H358 A549, H1299 SPC-A1, SCC-35 SW900, HCC827	EGFR, MET β-catenin ERK1/2-GSK3β PI3K-Akt c-Src/AKT	Eggermont et al. (2023) Jang et al. (2011) Li et al. (2018a) Zhong et al. (2015) Lee et al. (2018)
Liver cancer	c-CBL	SMMC-7721, MHCC97H Huh-7, Hep3B	AP-1/NF-AT1	Zhang et al. (2015a) Jiang et al. (2017)
Pancreatic cancer	c-CBL CBL-b CBL-c	HEK293T, PaCa-2 SW1990 Patu8988, Bxpc-3 Aspc-1 Jr305, panc-1, panc-28 Capan-1/2	PI3K/AKT Erk EGFR c-Met/Crk/JNK NF-κB Met	Donahue et al. (2012) Song et al. (2020) Jang et al. (2020) Chekmarev et al. (2021) Wesley et al. (2018) Hill et al. (2010)
Gastric cancer	c-CBL CBL-b CBL-c	MKN45, MKN74 HGC-27 MGC803 SGC-7901 AGS SNU-1	EGFR PI3K/Akt MAPK/ERK miR-940/CBL-b/STAT5a ERK1/2 Met JWA/c-CBL/HER2	Dong et al. (2010) Qu et al. (2009a) Qu et al. (2011) Fan et al. (2018) Qi et al. (2019) Lai et al. (2012) Deng et al. (2020)
Colorectal cancer	c-CBL CBL-b	HCT116, SW480 HT29, Caco-2	EGFR Wnt/β-catenin	Wang et al. (2012) Kumaradevan et al. (2018)
Renal cancer	c-CBL	769-p, A-498 ACHN, CAKI-1 OS-RC-1	Met EGFR Wnt/β-catenin	Maroun and Rowlands (2014) Sahu et al. (2022) Chitalia et al. (2013)
Prostate cancer	c-CBL CBL-b	LNCap, PC3 DU145, C4-2B	EGFR Erk1/2	Kitai et al. (2017) Shrestha et al. (2018)
Thyroid cancer	c-CBL	IHH-4 K1, TPC-1	Wnt/β-catenin circ-ITCH/miR-22-3p/CBL	Guo et al. (2021) Guo et al. (2021)
Melanoma	c-CBL CBL-b	MEL202 MMAc-SF	FAK-GRB2-SRC PD-L1/PD-1	Nihal and Wood (2016) Fujiwara et al. (2017)
Ovarian cancer	c-CBL	SKOV3 A2780	EGFR RAS-MAPK	Hanson et al. (2014) Hanson et al. (2014)
Breast cancer	c-CBL CBL-b CBL-c	MDA-MB-231, mSKBR-3 MCF-12A, MDA-MB-231 HBL101, MDA-MD-435 HS598TMCF-10A MCF-7, BT-20 MCF-10F, AU565 ZR-75-1, BT-483, BT-474	EGFR, RANKL/RANK Src-Akt/ERK PI3K/Akt, MAPK/Erk PI3K-PKB-GSK-3	Flanders et al. (2003) Zhang et al. (2015b) Li et al. (2018c) Chen et al. (2018)

### 3.1 Glioma

Glioma is a common primary central nervous system tumor with complex pathogenesis, accounting for 81% of malignant brain tumors. There is a significant level of mortality and morbidity associated with these conditions, despite their rarity (Wu et al., 2023). Inhibition of CBL can significantly inhibit the formation of glioma cells. Previous research has been observed that c-CBL mediates the migration and invasion of glioma cells, such as C6 and human A172 glioma cells, by exerting a negative regulatory effect on αPix. The findings indicate that c-CBL exon jumping can contribute to the development of human glioma and its malignant characteristics (Seong et al., 2014).

In 2013, the study of Jingwen Zhang revealed that tissue transglutaminase (TTG) blocks the EGFR ubiquitination catalyzed by c-CBL through the interaction with c-CBL, which enhances EGFR signaling and thus promotes the formation of glioma (Zhang J. et al., 2013). The overexpression of EGFR is considered to be linked to the malignant phenotype of human glioblastoma (GBMs) (Hisano, 1976; Katayama et al., 1999). The association between CBL and EGFR offers a new way to study glioma. Ephrin ligands play a crucial role in the development of the nervous system. In the initial studies, it was observed that the expression of ephrinA5 was markedly reduced in primary gliomas when compared to normal tissues. The enhanced expression of ephrinA5 may reduce the carcinogenicity of human glioma U373 cells. EphrinA5 enhanced the binding of c-CBL to EGFR (Li J. J. et al., 2009). Zinc finger CCCH-type containing 15 (ZC3H15), a highly conserved protein that plays a role in various cellular processes, has been implicated in tumorigenesis. It facilitates the proliferation, migration, invasion, and tumorigenesis of GBM cells by triggering the activation of the EGFR signaling pathway. The findings demonstrated that ZC3H15 suppressed the transcription of CBL. When a specific proportion of CBL was silenced, it eliminated the inhibitory impact of ZC3H15 knockdown on the proliferation, migration, and invasion of GBM cells (Hou et al., 2022).

Within the cellular pathway, glioblastoma (GBM) cells employ Cool-1/β-pix to obstruct the regular activation of the c-CBL ubiquitin ligase through the redox/Fyn/c-CBL pathway. Also, the suppression of c-CBL plays a significant role in GBM cell functionality. In an *in vivo* setting, the knockdown of Cool-1 substantially impedes the tumor-forming capabilities of GBM cells, which is closely associated with the alterations in c-CBL (Stevens et al., 2014). The adaptor protein STA/CIN85/Ruk plays a role in regulating multiple signal transduction pathways, including the internalization of tyrosine kinase receptors through CBL ubiquitin ligase and the modulation of PI3K activity through interaction with its subunits of regulation. In a direct analysis conducted using an electrochemical substrate impedance sensor (ECIS), it was observed that c-CBL decreased cellular adhesion (Schmidt et al., 2003). A 2015 Ning Bu study found out that dendritic cell-derived exosomes (DEXs) were produced by loading dendritic cells with the GL261 glioma cells containing chaperone protein-rich cell lysates (CRCLs). DEX-DCs was negative for the CBL-b and c-CBL signaling pathways, which leads to the activation of the PI3K/Akt and extracellular signal-regulated kinase (ERK) signaling pathways in T cells. The results show that its antitumor effects can be exerted by regulating CBL-b and c-CBL signaling pathways (Bu et al., 2015). PTPRU knockdown also led to the increase of tyrosine pY in c-CBL, which further affects β-catenin and focal adhesion signals, and promotes the progression of glioma. It can be confirmed that CBL provides a new target for the treatment of brain glioma.

In recent years, as research deepens, more and more ways of CBL affecting glioma have been discovered. MiR-30e enhances cell invasion, which involves the stimulation of EGFR expression and the subsequent activation of its downstream signaling mediators, such as AKT and ERK. As confirmed by the analysis, CBL-b was the immediate target of miR-30e (Kwak et al., 2015). Furthermore, glioma cell lines exhibit a robust antitumor response to the apoptosis-inducing ligand (TRAIL) related to tumor necrosis factors. The addition of TMZ also increased the expression of c-CBL. The results showed that the inhibition of c-FLIP expression can be achieved by TMZ through the degradation of ubiquitin through c-CBL-mediated ubiquitination, thereby overcoming TRAIL resistance (Zhitao et al., 2015). In 2020, it was found out that dequalinium chloride (DECA) can significantly inhibit the growth and proliferation of glioma cells. DECA may enhance glioma cell apoptosis by influencing the expression of CBL gene (Yu et al., 2020). These discoveries have presented endless possibilities for the treatment and cure of glioma.

### 3.2 Lung cancer

Lung cancer is a leading cause of cancer-related mortality on a global scale. The predominant type of lung cancer, known as non-small cell lung cancer (NSCLC), accounts for the majority of diagnosed cases (Parma et al., 2022; Attieh et al., 2023; Lin et al., 2023). As our understanding of the molecular mechanisms underlying lung cancer has advanced, personalized approaches have emerged that take into account specific molecular abnormalities. Notably, several biomarkers and therapeutic targets such as EGFR, K-ras, ALK, MET, CBL, and COX2 have gained prominence in this context. These molecular markers and targets hold a great potential in guiding treatment decisions and developing novel therapies for lung cancer (Salgia et al., 2011; Godoy et al., 2023). In a pioneering study, TKamei investigated the expression and tyrosine phosphorylation of the proto-oncogene product c-CBL in different human cancer cell lines and surgical specimens, with the aim to identify the signaling pathways linked to the development of human tumors. The findings underscore the significant role of c-CBL signaling in the process of human tumorigenesis (Kamei et al., 2000). An analysis of the publicly available microarray dataset obtained from the Gene Expression Omnibus (GEO) was conducted to investigate the critical differentially expressed genes (DEG) in non-small cell lung cancer (NSCLC). The study results show a noteworthy correlation that CBL has carcinogenic function, that is, the increased expression of CBL mRNA is associated with a significant improvement in the prognosis of NSCLC patients (Erkin et al., 2022).

CBL plays a crucial role in a negative modulation of the epidermal growth factor receptor (EGFR) signaling pathway in various organisms. It plays a pivotal role in the processes related to cell growth and development, and human pathology, including lung cancer. EGFR is a powerful oncogene, and EGFR activation mutations are the key determinants of carcinogenic transformation and therapeutic targets for non-small cell lung cancer. c-CBL has recently been found as a direct phosphorylated target of Pollo-like kinase 1 (PLK1) in tyrosine kinase inhibitor (TKI) -sensitive EGFR-mutant NSCLC cells, and PLK1 influences the stability of c-CBL in a manner dependent on its kinase activity. CBL functions by selectively directing activated ErbB receptors towards ubiquitination, thus facilitating the ligand-induced desensitization of EGFR. The connection between EGFR and c-CBL has been demonstrated to rely on the phosphorylation of the tyrosine residue 1045 within EGFR (Han et al., 2006; Eggermont et al., 2023). In the presence of oxidative stress, the phosphorylation of tyrosine 1045 on the epidermal growth factor receptor (EGFR) acts as the point of interaction with the ubiquitin ligase c-CBL. This interaction prevents EGFR from recruiting c-CBL and the subsequent ubiquitination and degradation (Goldkorn et al., 2005).

The Met receptor and its associated ligand, known as HGF, play a pivotal role in the signaling pathways implicated in the development of cancer (Hajmomeni et al., 2023). These pathways encompass the control of cell proliferation, invasion, angiogenesis, and the regulation of cancer stem cells. Furthermore, the modifications in the pathways governing Met, including the ubiquitin ligase c-CBL, can potentially trigger Met activation in carcinogenic contexts (Maroun and Rowlands, 2014). The identification of an *in vivo* electronic mutation of Met kinase that leads to an alternative splicing transcript involves the simultaneous deletion of a membrane domain. Consequently, this deletion causes the loss of binding capability for CBL E3 ligase (Kong-Beltran et al., 2006; Jang et al., 2011). In the case of lung cancer, MET gene amplification has been identified in a subset of adenocarcinomas. While the somatic mutations of MET are infrequent in lung adenocarcinoma, and nearly all reported mutations thus necessitate the alterations in splicing, deletions, and membrane domains to facilitate the binding with c-CBL E3 ligase. The mutations affecting the MET c-CBL binding site make up roughly 2% of all MET exon 14 alterations seen in lung cancer. It has been demonstrated that these mutations in the MET c-CBL binding site should be classified as a distinct subtype of MET exon 14 changes. The lung cancer patients who harbor such mutations should be considered for targeted therapy (Wiesweg et al., 2020; Fernandes et al., 2023).

Sprouty2 plays a crucial role in restraining cell proliferation and managing signal transduction processes. It was observed that the second phase of cell cycle-specific expression pattern of Sprouty2 relies entirely on the process of c-CBL ubiquitination. The level of Sprouty2 in cells is a consequence of highly coordinated interactions between Ras and c-CBL. When c-CBL as a recognized binding partner of Spry2 is forcibly expressed, it diminishes the levels of Spry2 protein. Conversely, tapping down c-CBL increases Spry2 protein levels. These findings suggest that CUG2 reduces the abundance of Spry2 protein, which acts as a negative regulator of cell proliferation, through c-CBL. This reduction may potentially activate the EGFR and β-catenin signaling pathways, thereby contributing to the emergence of cancer stem-like characteristics (Mayer et al., 2010; Yawut et al., 2020).

ShRNA targeting CBL-b exhibited different effects on cell behavior depending on cell type. Specifically, it stimulated the migration and invasion of lung adenocarcinoma cells A549 and H1975. Furthermore, CBL-b plays a regulatory role in modulating the expression of proteins involved in the PI3K and ERK1/2-GSK3β signaling pathways in both A549 and SW900 cells. Notably, the mRNA expression of CBL-b varies between lung adenocarcinoma and squamous cell carcinoma. These differences in signaling pathways may contribute to the distinct correlation observed between CBL-b expression and survival outcomes in these two types of lung cancer (Chang et al., 2013; Li P. et al., 2018). Pyk2, a proline-rich tyrosine kinase 2, plays a crucial role in regulating cell adhesion and detachment. In this context, CBL-b interacts with Pyk2 to downregulate its expression, thus facilitating the trypsin-induced degradation of Pyk2. These intriguing findings indicate that CBL-b promotes cell detachment by inducing the monoubiquitination of Pyk2 (Padrón et al., 2007; Puri et al., 2007; Shtiegman et al., 2007; Fan et al., 2014).

A study investigating the clinical relevance of miR-1323 in lung cancer patients focused on its prognosis and potential mechanism. The results revealed that miR-1323 was found to enhance the migration of lung adenocarcinoma (LUAD) cells by suppressing the expression of CBL-b. Importantly, the high expression of miR-1323 was associated with a poorer prognosis in LUAD patients. Conversely, the high expression of CBL-b was indicative of a better prognosis in the patients with non-small cell lung cancer (NSCLC) and LUAD (Zhao H. et al., 2020). CBL wild-type cells exhibited lower MET expression levels when compared to CBL mutant cells. Additionally, the ubiquitination of MET diminished in CBL mutant cells compared to wild-type cells. These findings suggest that CBL may serve as a potential positive indicator for MET-targeted therapy in non-small cell lung cancer (NSCLC) (Tan et al., 2017).

Following the treatment with the DNA methylation inhibitor 5′-nitropyrimidine, the expression of CBL-c, an epigenetic demethylation target, can be enhanced in non-small cell lung cancer (NSCLC). It is indicated that 5′-nitropyrimidine treatment has the potential to upregulate CBL-c expression in NSCLC by targeting DNA methylation processes. In xenograft models, the reduction of CBL-c expression had a significant impact on the viability and clonability of cells *in vitro*, leading to suppressed tumor growth. Furthermore, silencing CBL-c expression sensitized the NSCLC cells with EGFR mutations to the treatment with tyrosine kinase inhibitors, thus enhancing their therapeutic effectiveness. Hence, targeting CBL-c is a promising strategy for inhibiting tumor growth and improving the response to tyrosine kinase inhibitor treatment in EGFR-mutated NSCLC.

The polyubiquitination facilitated by CBL-c plays a crucial role in promoting the preferential recycling of EGFR to the plasma membrane or its trafficking into the nucleus. Immunohistochemistry (IHC) analysis revealed a positive correlation between phospho-EGFR levels and CBL-c expression in lung adenocarcinoma. These findings highlight CBL-c as a potential diagnostic biomarker and suggest its potential as a novel therapeutic target for the treatment of non-small cell lung cancer (NSCLC). By targeting CBL-c, it may be possible to disrupt EGFR recycling and enhance lysosomal degradation, thereby inhibiting EGFR activation and potentially limiting tumor growth in NSCLC (Hong et al., 2018).

As a significant adapter protein with multiple domains, intersectin-1s (ITSN-1s) plays a crucial role in the development of various lung diseases. The restoration of ITSN-1s protein levels promotes the interaction between CBL E3 ubiquitin ligase and Eps8, leading to the increased ubiquitination of Eps8 tumor protein. As a result, the migration and metastasis of lung cancer cells (LC cells) are reduced (Drake-Lee and Morgan, 1989). Recently, it is evidenced that NSCLC-associated mutant EGFRs exhibit altered intracellular trafficking and reduced lysosomal downregulation as mediated by CBL ubiquitin ligase (Chung et al., 2014).

The C-terminal region of c-CBL engages with the cytoplasmic tail of PD-1. Then, c-CBL mediates the degradation of PD-1 via ubiquitination and proteasomal pathways, which is a process contingent upon the RING finger functionality of c-CBL. These findings indicate that c-CBL functions as an E3 ligase for PD-1, thereby acting as a regulator within the tumor microenvironment. These roles represent the previously undiscovered facets of tumor-suppressive activity performed by c-CBL (Lyle et al., 2019). The use of anti-PD-1/PD-L1 therapy has demonstrated a remarkably high clinical efficacy in the treatment of non-small cell lung cancer (NSCLC). In various wild-type EGFR cell lines such as A549 and H460, the ubiquitin ligases CBL-b and c-CBL have been found to inhibit PD-L1 expression by inactivating the signaling pathways involving STAT, AKT, and ERK. Moreover, there is a negative correlation present between the expression of CBL-b/c-CBL and PD-L1 in NSCLC tissues. These findings suggest that CBL-b and c-CBL possess novel regulatory mechanisms that influence PD-L1 expression in wild-type EGFR NSCLC cell lines (Wang et al., 2018).

Ring-sh2grb2 is a fusion protein consisting of the RING domain of CBL, which imparts E3 ligase activity, and the SH2 domain of Grb2, which acts as an adapter for EGFR. The ability of fusion proteins to facilitate EGFR ubiquitination and subsequent degradation provides a novel strategy for reducing EGFR signaling, which is a key process implicated in the growth and progression of lung cancer (Waterman et al., 2014; Zhong et al., 2015). The phosphorylation of Y845 has emerged as a significant event in cancer cells, while the phosphorylation of Y1045 is known to be associated with the ubiquitination and degradation facilitated by CBL. Targeting CBL specifically could have implications for oncogene addiction and its impact on STAT5, revealing the distinctive traits of the L858R EGFR signaling circuit that contribute to drug sensitivity and clinical effectiveness. These findings suggest that the precise modulation of CBL activity could provide a potential therapeutic strategy for exploiting oncogene addiction mechanisms and optimizing the outcomes of treatment for the patients with EGFR-mutant lung cancer (Kim et al., 2015). The reduced expression of c-CBL in human lung cancer tissue has been linked to the activation of c-Src/AKT signaling, increased cancer metastasis, and unfavorable patient survival. These findings suggest that c-CBL may function as a tumor suppressor by counteracting the potent carcinogenic signals mediated by c-Src (Li et al., 2016; Lee et al., 2018).

Previous studies have shown that EGFR-TKI icotinib has the ability to enhance the expression of CBL-b. The treatment with icotinib results in the increased levels of protein and the mRNA levels of CBL-b. In order to further investigate the role of CBL-b in the treatment of icotinib, a CBL-b knockout experiment was conducted. The CBL-b knockout assay found that the lack of CBL-b in PC9 and HCC827 cells reduced their sensitivity to icotinib, suggesting that CBL-b plays a crucial role in mediating the anti-proliferative effects of icotinib. In addition, CBL-b knockout partially restored AKT activation, and the icotinib treatment in PC9 cells inhibited AKT activation. Notably, the overexpression of p65, a major member of the NF-κB family of transcription factors (Zhu et al., 2023), reversed the upregulation of CBL-b as induced by icotinib. These findings collectively suggest that the icotinib-induced upregulation of CBL-b is mediated by the inhibition of NF-κB, and that CBL-b plays a role in the sensitivity of EGFR-mutated NSCLC cells to icotinib. Importantly, this study highlights that the low expression of CBL-b could potentially serve as a significant barrier to the efficacy of icotinib therapy (Zhang et al., 2019).

CBL-b interacts with Focal adhesion kinase (FAK) and facilitates the trypsin-induced ubiquitin-lysosomal degradation of FAK. These discoveries offer new perspectives on the roles of FAK and CBL-b in the process of cancer cell shedding (Fan et al., 2016). Icotinib, a highly specific EGFR tyrosine kinase inhibitor (EGFR-TKI), has demonstrated notably high clinical effectiveness and safety performance for the patients with non-small cell lung cancer (NSCLC) (Liu S. M. et al., 2023). Research has indicated that icotinib hinders the phosphorylation of EGFR, Akt, and extracellular signal-regulated kinases, while stimulating the expression of the ubiquitin ligase CBL-b (Mu et al., 2013).

Researchers have investigated the effectiveness of LY2801653, a potent inhibitor that targets both MET and RON tyrosine kinase receptors, which are known to be overexpressed in non-small cell lung cancer (NSCLC). The evaluation of LY2801653 showed promising results, as it effectively inhibited the activity of MET and RON, leading to a decrease in phosphorylation levels of CBL, PI3K, and STAT3 signaling molecules. This small molecule approach showed its potential in treatment for NSCLC (Kawada et al., 2014). *In vitro* and *in vivo* studies have demonstrated that suppressing the expression of the E2 ubiquitin conjugate CDC34 effectively hinders the proliferation of non-small cell lung cancer (NSCLC) cells. CDC34 competes with c-CBL to bind to Y1045, preventing the polyubiquitination and degradation of epidermal growth factor receptor (EGFR) (Zhao X. C. et al., 2020).

β-elemene, an active ingredient found in natural plant-based antineoplastic agents, has been studied for its potential in treating cancer. Notably, inhibiting the activation of Akt and ERK pathways enhances the apoptotic effects of β-elemene, indicating their involvement in the apoptosis induced by β-elemene in lung cancer cells. Additionally, the expression levels of c-CBL and CBL-b were found to be upregulated, suggesting the involvement of CBL-mediated regulation of Akt and ERK signaling in the apoptosis induced by β-elemene in lung cancer cells (Li et al., 2011). Shikonin (SK) is a naturally derived antitumor compound belonging to the naphthoquinone family. Upon the treatment with SK, there is a substantial increase in the expression of CBL-b, which gradually reaches its maximum level at 24 h. The downregulation of phosphorylated ERK (p-ERK) and the induction of apoptosis in NCI-H460 cells caused by SK were reversed when a CBL inhibitor Ps341 was used. These findings indicate that CBL-b plays a role in enhancing the apoptotic effects of SK in lung cancer cells by inhibiting the ERK pathway. Furthermore, the CBL protein has been found to promote the apoptotic effects of Shikonin by exerting a negative regulation on the PI3K/Akt signaling pathway in lung cancer cells (Qu et al., 2015).

The analysis of kinomics data revealed the upregulation of STAT3 and PDGF pathways, along with the suppression of CBL signaling in squamous cell carcinoma (SCC). A separate study revealed a significant association between the proteasome degradation facilitated by c-CBL and the downregulation of CSF-1R mediated by TD-92, which is a novel derivative of erlotinib. These findings provide valuable insights for the advancement of combination immunotherapies in the treatment of non-small cell lung cancer (NSCLC) (Tan et al., 2019; Shih et al., 2021). In this study, a cross-tissue transcriptome-wide association study (TWAS) was performed using UTMAS. The analysis involved the aggregated statistics from a large cohort of 13,327 lung cancer cases and 13,328 controls. Additionally, the genetic expression data on 44 different human tissues, as provided by the Genotypic Tissue Expression (GTEx) project, were included to investigate the patterns of gene expression across various tissues. Among them, five new genes, including CBL, exhibited a significant association with the risk of developing lung cancer in both cross-tissue and lung tissue models (Zhu et al., 2021).

In EGFR-mutated lung adenocarcinoma (LAD), CBL-c was observed to actively regulate the activation of EGFR through the processes of K6 and K11 biubiquitination, thus contributing to its carcinogenic function. CBL-c plays a role in promoting mitotic entry and participates in the regulation of cell cycle progression by stabilizing AURKA through the process of ubiquitination in LAD. These findings suggest that targeting CBL-c, in combination with paclitaxel, could provide a viable option of treatment for the LAD patients who currently lack access to targeted therapies (Hong et al., 2022).

### 3.3 Liver cancer

Liver cancer is an aggressive cancerous growth that originates in the liver. It can be classified into two main categories: primary liver cancer and secondary liver cancer, also known as metastatic liver cancer. There are various treatment options for liver cancer patients, including surgery, chemoradiotherapy, interventional treatments, targeted therapies, immunotherapy, and various other therapeutic modalities. These diverse treatment methods aim to effectively manage liver cancer and improve patient outcomes. The initial research has indicated a correlation between a low peritumor CBL density and the elevated expression of EGFR in the surrounding liver tissue. In addition, down-regulation of CBL can significantly inhibit the migration and proliferation of HCC cells. The prognostic value was further enhanced when the assessment included peritumor CBL levels and EGFR expression, especially in the context of early HCC recurrence (Zhang J. B. et al., 2015).

In hepatocellular carcinoma (HCC), the expression of 14-3-3σ is elevated, and its overexpression is linked to aggressive clinicopathological characteristics and unfavorable prognosis. Moreover, 14-3-3σ actively promotes drug resistance and metastasis in HCC cells. It accomplishes this by interacting with EGFR and exerting a significant inhibitory effect on the degradation of EGFR induced by EGF. 14-3-3σ achieves this by inhibiting the association between EGFR and c-CBL, and by impeding c-CBL-mediated ubiquitination of EGFR subsequently (Song et al., 2021). Research has demonstrated that miR-22 exerts an inhibitory effect against tumor in hepatocellular carcinoma (HCC) through the regulation of various genes. Specifically, miR-22 indirectly modulates the expression of SPRY2 by targeting the E3 ligase CBL, which is associated with SPRY2. By inhibiting the expression of CBL, miR-22 enhances the stability of SPRY2 and then upregulates its levels. This regulatory mechanism leads to not only the inhibition of epithelial-mesenchymal transition (EMT), also a decrease in cell migration and invasion. Furthermore, miR-22 reduces the expression of hepatocellular carcinoma stem cell (CSC) marker genes (Cui et al., 2023).

The upregulation of miR-486-5p leads to a significant decrease in both mRNA and protein levels of CBL. Conversely, the overexpression of CBL counteracts the inhibitory effect of miR-486-5p on the proliferation and migration of hepatocellular carcinoma (HCC) cells. Notably, there exists a negative correlation between the expression of CBL and miR-486-5p in HCC. The downregulation of CBL by miR-486-5p contributes to the suppression of HCC cell proliferation and migration, which underscores the potential therapeutic significance of targeting this miRNA-CBL axis in HCC treatment (He et al., 2019).

Recent research has revealed the presence of a long non-coding RNA (lncRNA) called lnc-EGFR, which exhibits the specific binding to EGFR. This binding event hinders the interaction and subsequent ubiquitination of EGFR by c-CBL, thus stabilizing EGFR. This particular mechanism suggests that lnc-EGFR represents a potential therapeutic target for hepatocellular carcinoma (HCC), providing a solution to the intervention in HCC-related immune suppression (Jiang et al., 2017). Furthermore, it has been observed that the cytoplasmic protein c-CBL experiences substantial downregulation in the KYN-2 cells infected with MEK1, indicating that c-CBL could serve as a potential downstream mediator in the integrin/MEK/ERK signaling pathway associated with cell scattering. This signaling pathway emerges as a crucial molecular pathway underlying intrahepatic metastasis in human hepatocellular carcinoma (HCC), which highlights its significance to understanding and targeting HCC metastasis within the liver (Honma et al., 2006).

Notably, CBL has been identified as a key factor in the recruitment of 85 kDa (CIN85) proteins into the endocytic complex. Independent of the ubiquitin-ligase activity of CBL, this recruitment occurs in an EGF-dependent manner (Schroeder et al., 2010). Zinc finger CCCH type 15 (ZC3H15), a highly conserved protein implicated in different cellular processes associated with tumorigenesis, has emerged as a promising marker in hepatocellular carcinoma (HCC). Notably, ZC3H15 has been observed to exert inhibitory effects on the transcription of CBL, an E3 ubiquitin ligase that is degraded by the proteasome upon activation of the EGFR pathway. When CBL is silenced, it partially offsets the inhibitory effect of ZC3H15 gene knockout on cell proliferation, migration, and invasion (Shirasaki et al., 2022).

### 3.4 Pancreatic cancer

Pancreatic cancer is a prevalent malignancy affecting the digestive tract and it is often referred to as the “king of cancer” due to its aggressive nature. It represents one of the most lethal diseases in humans, with a dishearteningly low 5-year survival rate of less than 5%. The prognosis for pancreatic cancer remains poor, largely due to late-stage diagnosis, the limited range of effective treatment options, and the propensity for metastasis. KRAS mutations are frequently observed in pancreatic cancer and other types of cancer, making KRAS an appealing target for therapeutic interventions.

To tackle this challenge, researchers have developed several chimeric proteins by fusing the C-terminal regions of E3 ubiquitin ligases (such as CBL, CHIP, E6AP, VHL) and the viral infectivity factor (VIF) encoded by human immunodeficiency virus type 1 (HIV-1) with the Ras binding domain (RBD) of Raf. These engineered chimeric proteins have demonstrated the capability to induce the degradation of KRAS mutants and the cell death in KRAS mutant tumor cell lines. This breakthrough holds a potential in the treatment of various tumors with KRAS mutations or over-activation, including the cases of pancreatic cancer recurring after surgery (Pan et al., 2016). It was found that down-regulating CBL could significantly inhibit the formation of pancreatic cancer cells. Prior studies have revealed the potential dysregulation of SRC signaling in pancreatic ductal adenocarcinoma (PDAC) due to the alterations in CBL, where CBL acts as a pivotal molecule in modulating SRC and/or PI3K/AKT signaling. This is attributed to the capacity of CBL to function as a ubiquitin ligase, targeting such critical proteins as EGFR and SRC for ubiquitination and subsequent degradation. By acting as a regulatory “pin” molecule, CBL can exert control over the activity and expression of SRC, thereby influencing the downstream signaling pathways involved in cancer progression, including the PI3K/AKT pathway. The intricate interrelationship between CBL, SRC, and PI3K/AKT signaling highlights the potential significance of targeting these molecular interactions to the therapeutic interventions in PDAC (Donahue et al., 2012).

The insufficient levels of CBL have been identified as a factor contributing to the development of chemotherapy resistance in pancreatic ductal adenocarcinoma (PDAC), which is attributed largely to the stress-induced activation of EGFR (epidermal growth factor receptor). This activation of EGFR can be effectively countered by inhibiting EGFR, which restores chemosensitivity. The findings indicate that dysubiquitination plays a critical role in the overactivation of EGFR in PDAC. Moreover, the low levels of CBL can be taken as a defining characteristic of PDAC tumors that are likely to respond favorably to erlotinib therapy, a targeted drug that inhibits EGFR activity (Kadera et al., 2015). Hematopoietic progenitor kinase 1 (HPK1) enhances the interaction between the oncogenic receptor tyrosine kinase AXL and the CBL proto-oncogene (c-CBL). This interaction facilitates the promotion of AXL ubiquitination, thus leading to a decrease in AXL-mediated signaling pathways, such as phosphorylated Akt and phosphorylated Erk signaling. As a result, the invasion capacity of pancreatic ductal adenocarcinoma (PDAC) cells is reduced. This knowledge may contribute potential solutions to the therapeutic interventions targeting the HPK1-AXL-CBL axis for suppressed PDAC progression and metastasis (Song et al., 2020).

In addition, the GEM implant local delivery of gemcitabine inhibits tumor cell growth by promoting c-CBL-mediated EGFR degradation, thus inhibiting the proliferation, angiogenesis, and epithelial mesenchymal metastasis of pancreatic tumor (Hong et al., 2013; Jang et al., 2020). The expression of CBL-b in the patients with resectable pancreatic ductal adenocarcinoma (PDAC) serves as an independent predictor of unfavorable prognosis. Furthermore, the combination of CBL-b expression with pathological lymph node involvement (pN), histological differentiation, and CA19-9 levels enhances the risk stratification and prognostic evaluation in the individuals with resectable PDAC (Dong et al., 2017).

Growing evidence supports the role of microRNAs in regulating the proliferation of pancreatic ductal adenocarcinoma (PDAC) cells and their potential as prognostic indicators for PDAC outcomes. To identify the relevant microRNAs, a comparison was conducted using microRNA microarrays to analyze the expression profiles between the groups with good and poor prognosis. Among the identified candidates, microRNA-891b (miR-891b) emerged as a prognostic predictor for PDAC, which was validated in an initial group and subsequently evaluated as an independent predictor for the overall survival in a separate cohort of resectable PDAC patients. MiR-891b can specifically target the CBL-b gene, leading to an increase in the expression of the tumor suppressor protein p21. This increase in p21 expression plays a crucial role in inhibiting the proliferation of pancreatic ductal adenocarcinoma (PDAC) cells. By directly acting on the CBL-b gene, miR-891b provides a potential mechanism for suppressing PDAC cell growth and promoting a tumor-suppressive environment (Dong et al., 2016). MiR-29b-2-5p directly binds to the CBL-b gene, downregulates its expression, and reduces the CBL-b-mediated degradation of p53. The expression of miR-29b-2-5p in PDAC tissues showed a negative correlation with CBL-b. These findings suggest that miR-29b-2-5p improves the prognosis of PDAC by targeting CBL-b to promote p53 expression and is an important prognostic factor for PDAC (Li C. et al., 2018).

NDRG1, an important regulator of signaling pathways, has been implicated in various cancers, including aggressive pancreatic tumors. Recent research has revealed that NDRG1 possesses the ability to inhibit multiple tyrosine kinases. One of its potential targets is the c-CBL protein, which is known to regulate protein tyrosine kinases and receptor tyrosine kinases through the process of ubiquitination. By promoting the degradation of receptor tyrosine kinases, c-CBL can exert tumor-suppressive effects and inhibit cancer progression. However, it is noteworthy that c-CBL can also act as a mediator in oncogenic signaling pathways. In particular, it can function as a docking protein in the c-Met/Crk/JNK and PI3K/AKT pathways, thereby contributing to tumor development. This dual role of c-CBL highlights its complex involvement in cancer and suggests that its impact on tumorigenesis can vary depending on the cellular context and specific signaling cascades (Kales et al., 2014; Chekmarev et al., 2021).

Targeting negative regulators in the downstream signaling pathway of the T cell receptor (TCR) has been identified as a promising approach to enhance the efficacy of cancer immunotherapy. Among these regulators, CBL-b, an E3 ubiquitin ligase, plays a crucial role in governing the PI(3)K signal. Recent studies have revealed that the absence of CBL-b predominantly enhances the transduction of the NF-κB signal. In contrast to wild-type (WT) T cells, the deletion of CBL-b has been shown to significantly boost the functional responses of CD8^+^ T cells. These findings demonstrate that the absence of CBL-b can augment the overall functionality and cytotoxic potential of CD8^+^ T cells in response to TCR stimulation (Wesley et al., 2018).

The enhanced activation of the hepatocyte growth factor receptor (Met) signaling pathway has been associated with the pancreatic tumor initiation and unfavorable prognosis in patients. This heightened signaling contributes to tumor cell growth, survival, and viability. In the context of pancreatic Suit-2 cells, the impaired binding of CBL to internalized Met leads to the reduced ubiquitination of the receptor. The dysregulated interaction between CBL and internalized Met disrupts the normal process of receptor ubiquitination, which is critical to the regulation of cellular signaling. As a result, the extended presence of Met on the cell surface enables the prolonged activation of downstream signaling pathways, thus promoting the aggressive behavior of pancreatic tumor cells (Jäggi et al., 2008; Hill et al., 2010; Zhang et al., 2023).

### 3.5 Gastric cancer

Gastric cancer (GC) is a prevalent form of cancer globally, ranking as the fifth most common one. Despite a notable progress in the approach to screening and treatment, GC continues to pose significant challenges and remains a highly debilitating illness. The median survival rate for individuals with advanced-stage GC is approximately 14.2 months, emphasizing the urgent need for improved therapeutic options and strategies to enhance patient outcomes. The efforts to develop novel diagnostic methods, targeted therapies, and personalized treatment approaches are crucial for tackling the complex nature of GC and improving survival rates for affected individuals (Wang et al., 2024). In this study, the immunohistochemical method was used to measure the expression levels of c-CBL, CBL-b, and EGFR in 124 gastric cancer tissue samples and 16 normal gastric mucosa samples. The findings revealed that the positive rates of c-CBL, CBL-b, and EGFR in the gastric cancer group were significantly higher compared to the normal group (71.0% vs. 18.0%). Moreover, the overexpression of c-CBL, CBL-b, and EGFR exhibited a significant association with the invasion and progression of gastric cancer. These results suggest that c-CBL and CBL-b have carcinogenic functions and may become novel molecular markers for gastric cancer (Dong et al., 2010).

c-CBL is a key negative modulator of cell signal transduction, and its role has attracted widespread attention for the pathogenesis and regulation of human cancer. We examined the levels of c-CBL protein expression and tyrosine phosphorylation in various human tumor cell lines and surgical specimens. The results showed that the tyrosine phosphorylation of c-CBL protein occurred in a tumor-specific manner in 12 (33%) of the analyzed tumor tissues. These findings highlight the importance of c-CBL signaling in human tumorigenesis, underscoring its potential involvement in the development and progression of stomach cancer, as well as other cancer types concerned in this study (Kamei et al., 2000). Furthermore, a research was conducted to collect a total of 84 gastric cancer tissues comprised of 44 gastric cancer tissues and 40 adjacent normal tissues. Immunohistochemistry (IHC) analysis was performed on tissue microarrays (TMA) to examine the expression of the c-CBL protein. The results showed that the expression level of c-CBL in gastric cancer tissues was significantly lower compared to the adjacent normal tissues, with a low expression rate of 61.4% (27/44) in gastric cancer cases. This finding indicates a downregulation of c-CBL in gastric cancer. Overall, these findings highlight the potential role of c-CBL in gastric cancer pathogenesis and provide insights into the dysregulation of c-CBL and its associated molecules in gastric cancer progression (Li Y. et al., 2009).

The incidence of diffuse EGFR expression was found to rise in tandem with the increased CBL expression levels. Notably, the application of transforming growth factor α exhibited a substantial capacity to induce the expression of CBL protein within gastric cancer cells. These observations suggest a potential linkage between CBL and the EGFR system, which may be implicated in the process of tumor initiation, invasion, and metastasis. Consequently, CBL could emerge as a promising novel molecular marker for identifying invasive gastric cancer (Ito et al., 2004). G protein pathway inhibitor 2 (GPS2) is widely expressed in human tissues, including the stomach. The elevated GPS2 levels have been demonstrated to curb the proliferation, colony formation, tumorigenesis, and invasion of gastric cancer cells. Furthermore, GPS2 facilitates EGFR ubiquitination by benhancing its interaction with the E3 ligase c-CBL, which induces the subsequent degradation via the lysosomal pathway, thus destabilizing EGFR protein (Si et al., 2021).

In the context of exosome-induced apoptosis, there was an observable upregulation in the expression of CBL-b and c-CBL. It appears that the ubiquitin ligases belonging to the CBL family may play a role in mediating certain effects of caspase activation (Qu J. L. et al., 2009). Furthermore, arsenic trioxide (ATO) has been observed to induce cell cycle arrest in a variety of solid tumors. ATO exerts an upregulatory effect on the expression of CBL protein within cells. Notably, when CBL is inhibited by the proteasome inhibitor Ps341, there is a reduction in the apoptosis of NB4 cells. As revealed by these experiments, the inhibition of CBL plays a role in mediating ATO-induced apoptosis in NB4 cells and G2/M phase arrest in gastric cancer cells. CBL is postulated to achieve these effects by modulating the p53-activated PI3K/Akt pathway (Li Y. et al., 2009). IFN-α has the capability to suppress the expression of the c-CBL family. Additionally, c-CBL acts as an inhibitor of IFN-α-induced ERK activation. The experimental results indicate that IFN-α upregulates the MAPK/ERK pathway by downregulating c-CBL. This, in turn, augments the apoptosis of gastric cancer cells induced by TRAIL to a certain extent (Qu et al., 2011; Xu et al., 2012).

In prior research, it has been established that the expression levels of the ubiquitin ligase CBL-b are notably lower in multidrug-resistant (MDR) gastric cancer cells when compared to their parental counterparts. The overexpression of CBL-b has demonstrated the capacity to curtail the proliferation of MDR gastric cancer cells effectively, thus inhibiting tumor growth. Furthermore, the heightened expression of CBL-b enhances the interaction between CBL-b and IGF-1R. This interaction, in turn, induces the ubiquitination and subsequent degradation of IGF-1R, thus deactivating the IGF-1R pathway. These findings collectively suggest that the ubiquitin ligase CBL-b exerts an inhibitory effect on tumor growth in multidrug-resistant gastric cancer cells through the ubiquitination and degradation of IGF-1R (Barrueco et al., 1989; Xu et al., 2013; CHe et al., 2017). The enhanced expression of CBL-b has been observed to reduce cell migration in both *in vitro* and *in vivo* cultures of multidrug-resistant (MDR) cells. Furthermore, CBL-b overexpression effectively disrupts Epithelial-Mesenchymal Transition (EMT) by initiating the ubiquitination and subsequent degradation of EGFR. This disruption, in turn, hinders the EGFR-ERK/Akt-miR-200c-ZEB1 signaling axis. These findings collectively underscore the significance of CBL-b as a pivotal factor in the preservation of the epithelial phenotype and the inhibition of cell migration in MDR gastric cancer cells (Xu et al., 2017a).

CBL-b interacts with STAT5a, a member of the STAT protein family, thus leading to the degradation of STAT5a and the subsequent downregulation of PD-L1 expression. Notably, CBL-b is a target gene of microRNA miR-940 according to experimental data. The findings indicate that the miR-940/CBL-b/STAT5a axis intricately regulates the expression of PD-L1, thus promoting the proliferation and migration of gastric cancer cells (Fan et al., 2018). Furthermore, CBL-b exerts a positive regulatory effect on autophagy by suppressing the activity of mTOR (mammalian target of rapamycin) while enhancing the activation of ERK1/2 signaling pathway (Qi et al., 2019). The interaction between CBL-b and Pyk2 promotes Pyk2 degradation, which is facilitated by trypsin-induced Pyk2 degradation. The interaction between CBL-b and FAK is involved in this process, demonstrating that CBL-b promotes cell detachment through the ubiquitination of FAK (Fan et al., 2016).

Within the cellular pathway, CBL-b plays a pivotal role in reversing multidrug resistance (MDR) in gastric cancer by inhibiting the PI3K/Akt signaling pathway and down-regulating the expression of P-glycoprotein (P-gp). This modulation of CBL-b effectively counteracts the MDR phenotype in gastric cancer cells, contributing a potential therapeutic strategy to overcoming chemotherapy resistance (Zhang et al., 2009). The non-coding RNA UCA1 plays a regulatory role in the stability of GRK2 protein in gastric cancer (GC) cells. This is achieved by promoting the ubiquitination and subsequent degradation of GRK2, which involves CBL-c. This regulatory mechanism ultimately contributes to the promotion of metastasis in GC cells (Wang et al., 2017).

One of the key regulators of the duration of Met RTK signaling is the E3 ligase CBL. When the Met kinase is activated, CBL is recruited to the receptor and facilitates the ubiquitination, transportation, and subsequent degradation of the receptor. The aberrant signaling in MET-amplified gastric cancer cell lines has been identified as another mechanism contributory to abnormal signaling pathways. Dysregulation occurs through the loss of CBL protein, which depends on the activity of the Met kinase. This loss of CBL protein can be partially mitigated by lactase as the proteasome inhibitor. The absence of CBL not only disrupts CBL-mediated negative regulation but also leads to the liberation of other CBL targets, such as EGF receptors, from the signal dampening effects of CBL. Consequently, the MET-dependent loss of CBL induces crosstalk by indirectly enhancing EGF receptor signaling (Lai et al., 2012).

Receptor tyrosine kinases (RTKs) have been implicated in the initiation and progression of various cancers, including gastric cancer. Consequently, numerous inhibitors that target these RTKs have been developed and several of them have undergone clinical test. Notably, the amplification of specific RTKs such as c-Met, FGFR2, and ErbB2 is associated with the advancement of gastric cancer in humans. In these cases, their amplification leads to a loss of binding between CBL E3 ligase and the RTK, which diminishes ubiquitination and delays the downregulation of the receptor (Asaoka et al., 2010). LncRNAs, especially the cytoplasmic lncRNA LINC01485, have been identified as actively involved in gastric cancer development. LINC01485 plays a role in gastric cancer progression by promoting tumor cell growth and migration. Its interactions with EGFR and c-CBL disrupt the normal degradation of EGFR, thus contributing to the enhanced EGFR signaling in gastric cancer cells (Zhou et al., 2020).

CBL protein provides a theoretical basis for the treatment of gastric cancer. In the context of anthracycline-induced apoptosis, the expression of CBL-b is increased in MGC803 gastric cancer cells. The overexpression of CBL-b enhances cytotoxicity and promotes the induction of apoptosis through anthracycline drugs. Studies have revealed that overexpression of CBL-b significantly inhibits the activation of ERK (Extracellular Signal-Regulated Kinase), while the CBL-b with a dominant-negative function (CBL-b DN) significantly enhances the activation of ERK and Akt (Protein Kinase B). The results indicate that CBL-b sensitizes gastric cancer cells to anthracycline drugs by activating the mitochondrial apoptosis pathway and modulating the ERK and Akt survival pathways. It is implied that CBL-b may serve as a potential therapeutic target to enhance the effectiveness of anthracycline-based treatments in gastric cancer (Qu X. et al., 2009).

Studies have demonstrated that celecoxib, a selective COX-2 inhibitor, can upregulate the expression of the ubiquitin ligase CBL-b. However, when CBL-b expression is knocked down, the combined effect of celecoxib and rapamycin is attenuated, indicating that CBL-b plays a role in mediating the enhanced anticancer activity of this drug combination. In summary, celecoxib has been found to up-regulate the expression of CBL-b, and CBL-b plays a role in the inhibition of Akt phosphorylation by celecoxib. Additionally, CBL-b contributes to the combined anticancer effect of celecoxib and rapamycin. These findings highlight the potential of CBL-b in mediating the therapeutic effects of celecoxib and its combined therapeutic approaches (Cao et al., 2015).

DR5, CBL-b/c-CBL, and TRAF2 form the complexes in TRAIL-resistant gastric cancer cells that target CBL-b and c-CBL by inhibiting the interaction of TRAF2 with caspase-8 and subsequent caspase-8 multiubiquitination. The results showed that DR5-CBL-b/c-CBL-TrAF2 complex inhibited the TRAIL-induced apoptosis of gastric cancer cells by promoting TRAF2-mediated caspase-8 polyubiquitination (Xu et al., 2017b). Oxaliplatin can also promote the TRAIL-induced apoptosis of gastric cancer cells (Xu et al., 2009). Recent studies have shown that JWA is a biomarker of rapatinib in gastric cancer cells. JWA induces HER2 degradation in lapatinib-sensitive and resistant GC cells through c-CBL. The knockdown of c-CBL can salvage JWA-induced HER2 downregulation and lapatinib resistance. JWA/c-CBL/HER2 pathway plays a major role in lapatinib resistance (Ma et al., 2016). As confirmed in other studies, β-emolene upregulates CBL-b by inhibiting the expression of miR-1323. By regulating the miR-1323/CBL-b/EGFR signaling axis, β-elemene inhibits the metastasis of multi-drug resistant gastric cancer cells (Deng et al., 2020).

### 3.6 Colorectal cancer

Colorectal cancer (CRC) is a prevalent cancer affecting the gastrointestinal tract. Despite the advancements in early detection methods, it continues to be the third leading cause of cancer-related deaths globally. The primary reason for this is that more than 50% of patients eventually develop metastatic disease, which significantly impacts their prognosis, thus resulting in poor outcomes. Hence, it is imperative to enhance our understanding of the molecular basis of early colorectal cancer progression to metastasis. In order to gain a deeper understanding of the significance of c-CBL in colorectal cancer, we studied c-CBL expression levels in 22 patients with primary CRC. The findings revealed that in 8 out of the 22 CRC cases, there was an observable overexpression of c-CBL in the tumor samples. Early studies have confirmed that CBL has carcinogenic function and may be involved in the progression of gastrointestinal tumors (Calin et al., 1998). In addition, the recruitment of CBL through the interaction of Nox organizer 1 (NoxO1) and growth receptor-binding protein 2 (Grb2) leads to the reduced stability of NoxO1 stimulated by epidermal growth factor (EGF). This in turn suppresses the production of reactive oxygen species (ROS). Excessive ROS production may promote carcinogenic signaling, thus driving the formation of colorectal tumors (Joo et al., 2016).

In human colon cancer cell lines, CBL demonstrated co-localization with CIN85 and MUC1. This co-localized MUC1/CIN85/CBL complex is suspected to play a significant role in the promotion and progression of colon cancer. Therefore, the increased expression of CBL in the early stages of colon cancer could potentially be associated with prognosis (Cascio and Finn, 2015). c-CBL is a negative regulator of colorectal cancer (CRC). Research has demonstrated that c-CBL possesses the ability to change the tumor microenvironment and impede the activity of the programmed cell death-1 (PD-1) protein. The C-terminal of c-CBL interacts with the cytoplasmic tail of PD-1. According to the ring finger function, c-CBL disrupts the stability of PD-1 through ubiquitination-proteasome degradation (Lyle et al., 2019). Both the partial deletion of c-CBL and the inactivation of tumor suppressive colorectal polyposis (APC) promote the occurrence and development of colorectal cancer (Zygmunt et al., 1988). Furthermore, CBL exhibits notable anti-tumor effects. In a colon cancer cell line model, the presence of ephrinA5 exerted an inhibitory effect on EGFR by facilitating c-CBL-mediated EGFR ubiquitination and subsequent degradation. Consequently, the outcomes of the study imply that ephrinA5 inhibits the progression of colon cancer by promoting the degradation of EGFR as mediated by c-CBL (Wang et al., 2012).

Sprouty2 (Spry2) reduces protein degradation by binding to c-CBL, thereby increasing the expression of epidermal growth factor receptor (EGFR), and it is a potential biomarker for predicting anti-EGFR response in colon cancer patients (eng et al., 2010). In the patients with colorectal cancer (CRC) and poor prognosis, protein tyrosine phosphatase receptor type O (PTPRO) exhibits downregulation. This downregulation induces the activation of SRC and the phosphorylation of c-CBL ubiquitin ligase at Y845 and Y731 sites. The phosphorylation of c-CBL promotes its degradation, which in turn enhances the stability of EGFR (Asbagh et al., 2014). Studies have shown that the lack of CBL-b can overcome endogenous CD8^+^ T cell failure, and the lack of CBL-b can prevent CAR T cells from failure (Kumar et al., 2021). Recent studies have identified it as a potential linc-ROR target. By inhibiting the generalization and degradation of EGFR signals, linc-ROR acts as a tumor promoter (Chen et al., 2022).

Meanwhile, crcc-ccp-y371h mutants showed enhanced Wnt/β-catenin signaling pathways, increased Wnt target genes, and promoted angiogenesis and CRC tumor growth. The study confirmed a strong association between c-CBL and overall survival in colorectal cancer patients (Kumaradevan et al., 2018). Enhanced CBL activity leads to the downregulation of EGFR expression and the inhibition of colon tumor cell proliferation (Wang et al., 2013). Cortatin (CTTN) is overexpressed in a variety of tumors, including head and neck squamous cell carcinoma and colorectal cancer (CRC). Besides, it can be used as a biomarker for tumor metastasis. *In vitro* experiments demonstrated that CTTN promoted cancer cell proliferation, while the *in vivo* experiments conducted using CRC tumor xenografts showed enhanced tumor growth. Notably, CTTN expression impedes the ubiquitin-mediated degradation of EGFR by inhibiting the conjugation of c-CBL to EGFR. CoIP experiments revealed the interaction between CTTN and c-CBL in CRC cells, thus inhibiting EGFR degradation (Zhang et al., 2017). Studies have shown that nuclear β-catenin is negatively correlated with c-CBL. According to subsequent investigations, the loss of c-CBL function notably enhanced the proliferation of nuclear beta-catenin and colorectal cancer (CRC) tumors, as observed in both cell culture and mouse xenotransplantation models. In the final study, the function of c-CBL was confirmed as a negative regulator of CRC (Shashar et al., 2016).

To investigate the effect of polyphenol 2-(3, 4-dihydroxyphenol) ethanol (or hydroxytyrosinol, HT) in extra virgin olive oil on the expression of epidermal growth factor receptor (EGFR) in colon cancer cells and its mechanism. As confirmed by the results, HT mediated EGFR ubiquitination through the phosphorylation of CBL docking site pY1045, which led to the ubiquitination and degradation of the receptor. The findings of this research suggest some novel mechanisms that are essential for the prevention and treatment of colon tumors (Terzuoli et al., 2016).

### 3.7 Renal cancer

Renal cell carcinoma (RCC) is caused by the tubular epithelium of the kidney and represents one of the most common malignancies of the genitourinary system (Wang et al., 2023). To find the signaling pathways associated with human tumorigenesis, TKamei analyzed the expression and tyrosine phosphorylation of the proto-oncogene product c-CBL in various human tumor cell lines and surgical samples. The constitutive tyrosine phosphorylation of c-CBL protein was observed in human tumor cells, but not detected in control ECV304 cells. The surgical specimens of 36 human tumor tissues were studied, including 9 gastric cancer cases, 10 colon cancer cases, 6 kidney cancer cases, 2 liver cancer cases, 2 brain tumors, 2 uterine tumors, 1 breast cancer case, 1 thyroid tumor case, 1 bladder tumor case, and 2 lung cancer cases. The tyrosine phosphorylation of c-CBL protein was found to occur in a tumor-specific manner in 12 (33%) cases. The findings highlight the significance of c-CBL signaling to the initiation and progression of human tumorigenesis (Kamei et al., 2000).

A large number of studies have found that the high expression of CBL can promote the proliferation of renal cancer cells. The dysregulation of micrornas (mirnas) can lead to cancer, and early studies have found out that CBL is a direct target of miR-200a-3p. In RCC tissue samples, miR-200a-3p was negatively correlated with CBL. Through both *in vitro* and *in vivo* experiments, it has been demonstrated that the introduction of miR-200a-3p into renal cell carcinoma (RCC) cell lines results in the inhibition of cell proliferation and migration, along with the promotion of apoptosis. In summary, the miR-200a-3p/CBL regulatory axis is a novel pathogenesis of RCC and may be a candidate biomarker as well as therapeutic target for RCC (Ding et al., 2018). Met receptor tyrosine kinase (RTK) is an attractive target for tumor therapy. Met and its ligand HGF are crucial to the signaling pathways that participate in various essential cellular events such as cell proliferation, invasion, angiogenesis, and the regulation of cancer stem cells. The changes in pathways governing the activity of Met, including the ubiquitin ligase c-CBL, can lead to the activation of Met in the context of carcinogenesis (Maroun and Rowlands, 2014).

Mediated by small inhibitory rna, endogenous human SPRY2 (hSPRY2) silencing can eliminate the antiapoptotic effect of serum on adrenal cortical adenocarcinoma (SW13) cells. When hSpry2 is silenced, the tyrosine kinase binding domain (CBL-TKB) of c-CBL prevents the downregulation of such growth factor receptors as EGFR and maintains the antiapoptotic effect of the serum. Additionally, when Spry2 was silenced in the cells deficient in c-CBL, it was observed that the ability of serum to promote cell survival remained unchanged. Studies have shown that c-CBL is essential for the regulation of endogenous apoptosis mediated by HSPRy2, and hSPRY2 can isolate c-CBL, thereby enhancing the transduction of signals through growth factor receptors (Edwin and Patel, 2008).

Clear cell renal cell carcinomas (ccRCC) commonly exhibit the somatic mutations or reduced expression of von Hippel-Lindau (VHL) tumor suppressor factors. These alterations in VHL are considered as one of the causes of ccRCC. In VHL-deficient ccRCC cells, the downregulation of c-CBL indicates that c-CBL and pVHL downregulate activated EGFR collectively. The results suggest that pVHL restricts EGFR signaling by promoting the non-receptor polyubiquitination activation of c-CBL, which may lead to its degradation by the proteasome (Zhou and Yang, 2011). Nephroblastoma is the most prevalent malignant kidney tumor in children aged 3–4 years. Its development is attributed to the genetic changes in oncogenes (OG) and tumor suppressor genes (TG). Wilms tumors are associated with OG- and -TG. Through an analysis on the mRNA expression levels of 16 OGs and 20 TGs involved in crucial signaling pathways, the expression levels of PI3K and RAS in 24 fresh Wilms tumors were studied. A significant upregulation of CBL expression was observed after an extensive investigation (Chitalia et al., 2013; Sahu et al., 2022). These findings contribute innovative solutions to the treatment of kidney cancer.

### 3.8 Prostate cancer

Prostate cancer is an epithelial malignancy that occurs in the prostate. The expression of CBL can induce the apoptosis of prostate cancer cells and has the effect of inhibiting cancer. Earlier studies have found out that the reduced internalization of PC3-AR cells is related to a defect in the interaction between EGFR and two adaptive proteins that mediate endocytosis, namely, Grb2 and c-CBL. As a result of this reduced interaction, the ubiquitination of the receptor, which is primarily mediated by c-CBL, is also altered (Bonaccorsi et al., 2007). A novel study has identified the transcription factor TEAD1 and the ubiquitin ligase c-CBL as new markers for basal cells. These markers provide valuable insights into the characterization and understanding of basal cell populations (Knight et al., 2008).

In addition, the adenovirus infection expressing c-CBL shrnas resulted in the elevated levels of death receptor 4 (DR4) and DR5, both of which were responsible for the increase in TRAIL-induced apoptosis. It was further confirmed that the adenovirus expressed by c-CBL shrna could sensitize TRAIL-induced apoptosis both *in vitro* and *in vivo* (Kim et al., 2013). *In vitro* experiments have demonstrated that suppressing the expression of CBL-b can significantly enhance the function of T lymphocytes and their cytotoxic activity against prostate cancer cell lines, thus exerting a positive regulatory effect on anti-tumor immune responses (Zhou et al., 2014). Additionally, the strategy of gene silencing that targets CBL-b has been shown to enhance the immune function of T lymphocytes, increase their cytotoxicity against RM-1 prostate cancer cells, and significantly inhibit tumor growth in immune mice, suggesting its potential as a therapeutic approach. The study offers a potential immunotherapy strategy for prostate cancer (Shi et al., 2014).

In the cellular pathway, signal transduction adapter family member-2 (STAP-2) promotes tumorigenesis in prostate cancer cells by upregulating the EGF receptor (EGFR) signaling pathway. The inhibition of STP-2 significantly reduced the tumor growth of prostate cancer cell DU145. STAP-2 has been discovered to interact with EGFR and stabilize the receptor by inhibiting the c-CBL-mediated ubiquitination of EGFR (Kitai et al., 2017). CDKL3 is a recently discovered molecule that plays a regulatory role in human tumors. The most recent study unveiled a substantial upregulation of CDKL3 expression in prostate cancer tissues, which is positively correlated with the degree of tumor malignancy. These findings highlight the potential of CDKL3 as a therapeutic target in prostate cancer treatment. The downstream mechanism of CDKL3 may regulate STAT1 by inhibiting CBL-mediated STAT1 ubiquitination. Furthermore, CDKL3 has emerged as a novel promoter of prostate cancer and holds the potential as a therapeutic target for this disease, providing new ways of treatment development (Jiang et al., 2023).

Delta-catenin, a member of the armadillo protein subfamily called p120-catenin, has shown increased expression levels in advanced prostate cancer, suggesting its involvement in disease progression. It competes with c-CBL for binding to EGFR, which reduced c-CBL binding and then attenuate EGFR ubiquitination. Consequently, this mechanism increased the expression of membrane-bound EGFR and enhanced EGFR/Erk1/2 signaling. These results provide new insights into the role of delta-catenin in enhancing the prostate cancer mediated by EGFR/Erk1/2 signaling pathway (Shrestha et al., 2018; Brian et al., 2019).

Androgens and androgen receptors (AR) are involved in the early tumorigenicity and androgen refractory disease of prostate cancer (PCa). According to some data, the re-expression of AR in PCa cell lines provides a lower invasive phenotype. These effects include altering EGFR recruitment by binding Grb2 and c-CBL proteins and then reducing EGFR ubiquitination. The data showed that the crosstalk between genotypic and non-genotypic AR signals interfered with the ligand response signal of EGFR, thus reducing the aggressiveness of AR-positive PCa cells (Bonaccorsi et al., 2008). In addition, the study on the drug therapy of prostate cancer found out that demethoxycurcumin (DMC) has the most significant killing effect on prostate cancer PC3 cells. The compound DMC has been found to enhance the interaction between EGFR and CBL, thus inducing the tyrosine phosphorylation of CBL. These findings indicate that DMC exhibits potential antitumor effects on prostate cancer cells, suggesting its therapeutic utility in prostate cancer treatment (Hung et al., 2012).

### 3.9 Thyroid cancer

Thyroid cancer is the predominant form of malignancy affecting the thyroid gland, accounting for approximately 1% of all malignancies throughout the body. It encompasses various subtypes, including papillary, follicular, undifferentiated, and medullary cancers (Stevens et al., 2023). Valerio Costa employed targeted DNA sequencing techniques to uncover the novel missense mutations in genes such as CBL, NOTCH1, PIK3R4, and SMARCA4. Upregulated CBL can inhibit thyroid cancer.

This investigation involved the sequencing of genetic material from 50 patients with papillary thyroid carcinoma (PTC) and 30 healthy individuals. Additionally, another study unearthed the previously undocumented oncogenic mutations in thyroid cancer, encompassing various genes like BLM, CBL, CUTA, EP300, GSTM5, LMO2, PRAME, SBDS, SF1, TET2, TNFAIP3, XPO1, and ZRSR2 (Costa et al., 2015), totaling 13 oncogenic mutations. These mutations hold a potential as the target for future thyroid cancer treatments, offering a new solution to the development of novel anti-tumor drugs in the future (Pitt et al., 2016). Bioinformatic analysis and luciferase reporter gene assays demonstrated that circ-ITCH acts as a sponge for miR-22-3p, leading to the upregulation of CBL expression. The elevated CBL levels, in turn, exert an inhibitory effect on the activation of the Wnt/β-catenin signaling pathway, thus stalling the progression of papillary thyroid carcinoma (PTC) (Kamei et al., 2000).

Furthermore, it was discovered that circ-ITCH plays a pivotal role in impeding the progression of papillary thyroid carcinoma (PTC) through its influence on the circ-ITCH/miR-22-3p/CBL axis. Moreover, artificially elevating miR-22-3p levels is effective in suppressing CBL expression, while CBL siRNA significantly counteracts the upregulation of CBL induced by circ-ITCH. This intervention leads to the reduced proliferation of K1 and TPC-1 cells and an increase in apoptosis. Consequently, the establishment of the circ-ITCH/miR-22-3p/CBL axis signaling pathway provides a comprehensive explanation for the role of circ-ITCH in thyroid malignant tumors (Guo et al., 2021).

### 3.10 Melanoma

Melanoma, a highly aggressive and potentially fatal type of skin cancer, primarily affects the skin but can also be manifested in mucous membranes and internal organs. Melanoma accounts for approximately 3% of all tumor cases. A large number of studies have shown that CBL gene plays a crucial cancer-promoting role in immune regulation of primary melanoma and is closely related to the progression of metastatic disease (Erdrich et al., 2022). Research has indicated that the deficiencies in CBL-b can reverse the reduced IFN-γ production and impaired the tumor rejection observed in non-activated NKT cells. The resemblance between CBL-b (−/−) cells and NKT cells with a mutated RING finger domain highlights the crucial role of CBL-b E3 ligase activity in inducing functional impairment. CBL-b can bind to and enhance the process of CARMA1 monoubiquitination, which as a signaling molecule plays a crucial role in the activation of NFkappaB. The study reveals an essential signaling pathway that connects the CBL-b induced monoubiquitination with the activation of NFkappaB in NKT cells, which holds a potential in the development of effective strategies of cancer therapies for humans (Kojo et al., 2009).

Filamin A (FLNa) plays a significant role in intracellular receptor trafficking and has been involved in tumorigenesis. Furthermore, the absence of FLNa disrupts the interaction between EGFR and the ubiquitin ligase c-CBL (Hayes and Marrese, 1991). The expression of c-CBL, both at the mRNA and protein levels, was found to be significant in a group of human melanoma cell lines (A375, G361, Hs-294T, SK-Mel-2, SK-Mel-28, and 451Lu) through real-time fluorescent quantitative PCR and Western blot analysis. The clinical samples of melanoma and benign melanocyte nevus were also examined by means of Nuance multispectral quantitative imaging to determine the levels of c-CBL. It was observed that melanomas exhibited a range of immune responses to the c-CBL that overlapped with benign moles. To investigate the functional role of c-CBL in melanoma, c-CBL gene knockout was achieved through small interfering RNA (siRNA). The results showed that knocking out c-CBL led to a decrease in proliferation, clonal survival, and the migration of melanoma cells. This highlights the significant role played by c-CBL in the proliferation, migration, and invasion of melanoma cells, as well as the inhibition of the FAK-GRB2-SRC interaction.

These findings not only shed light on the importance of c-CBL in melanoma progression but also reveal its potential as a therapeutic target in inhibiting melanoma (Hinterleitner et al., 2012; Nihal and Wood, 2016). The deletion of Mirc11 reduces NK cell-mediated antitumor cytotoxicity but not to a significant extent. However, the loss of Mirc11 significantly reduced the production of pro-inflammatory cytokines *in vitro* and the interferon-gamma-dependent clearance of NK cells against B16F10-melanoma or *listeria* monocytogenes *in vivo*. CBL-b act as the ubiquitin-silenced modifier of Mirc11, which promotes the loss of Mirc11, thereby reducing the production of pro-inflammatory factors *in vitro*. Also, the interferon-γ-dependent clearance of NK cells is also reduced *in vivo* for *Listeria* monocytogenes or B16F10 melanoma (Nanbakhsh et al., 2019).

MiR-155 is downregulated in both malignant melanoma tissues and cell lines. Its ectopic expression has been shown to inhibit the migration and invasion of malignant melanoma cells. Interestingly, CBL has been identified as a novel target of miR-155. In malignant melanoma, CBL is negatively correlated with miR-155 levels. Additionally, CBL weakens the inhibitory effects of miR-155 overexpression on cell migration and invasion (Li et al., 2019). CBL-b is an attractive target for cancer immunotherapy as it plays a negative regulatory role in T cell activation. In the research conducted by Mai Fujiwara, the *in vivo* models of B16 melanoma were used to reveal a unique pattern of immunomodulatory resistance associated with CBL-b deficiency. This suggests that targeting CBL-b in cancer immunotherapy could provide a comprehensive approach to addressing multiple important “checkpoints” simultaneously (Fujiwara et al., 2017).

Proprion one (pro-PrP) is an adaptor protein for the E3 ligase c-CBL in human melanoma, allowing it to polyubiquitinate the activated insulin-like growth factor-1 receptor (IGF-1R), thus increasing melanoma metastasis. The glycosyl phosphatidyl peptide signaling sequence (GPI-PSS) is present in all human melanoma cell lines. The pro-PrP GPI-PSS sequence PVILLISFLI binds to c-CBL, and docks c-CBL with the inner cell membrane to produce a pro-PrP/c-CBL/IGF-1R trimer complex. Importantly, the synthetic peptide PVILLISFLI disrupted the PrP/c-CBL/IGF-1R combination *in vitro* and *in vivo*, thus reducing cancer cell autophagy and tumor aggressiveness (Li H. et al., 2022). Other researches have indicated that CBL-b is a degraded molecule that favors TreGs over Th9 cell development. CBL-b−/−TH9 cells exhibit stronger anticancer activity, which improves melanoma control *in vivo* (Schanz et al., 2021).

### 3.11 Ovarian cancer

Primary ovarian cancer accounts for 90%–95% of all malignant tumors that affect the female reproductive organs, while the remaining 5%–10% are secondary cancers that have metastasized to the ovaries (Harrison, 1987). Initial research has indicated that in ovarian cancer, CBL may function as a proto-oncogene involved in signal transduction and the regulation of tyrosine phosphorylation (Stanley et al., 1997). The studies involving overexpression have identified CBL-mediated EGFR ubiquitination as the primary mechanism responsible for ligand-induced EGFR downregulation. Furthermore, confocal immunolocalization studies have evidenced that CBL-dependent ubiquitination plays a pivotal role in the process of sorting EGFR from early endosomes to late endosomes/lysosomes during downregulation. These findings collectively establish CBL as the primary endocytic ligase responsible for EGFR degradation (Duan et al., 2003).

It has been discovered that the over-activation of the EGFR family of RTK, which includes EGFR (EGFR/HER-1 or ERBB-1), HER-2 (Neu or ERBB-2), HER-3 (ERBB-3), and HER-4 (ERBB-4), promotes the growth and progression of numerous tumor types. It is noteworthy that the overexpression of HER-2 is frequently linked to poor prognosis for a variety of cancers, including breast and ovarian cancer. By encouraging the ubiquitination and degradation of HER2, Xia Li worked out a design based on the chimeric ubiquitin ligase of CBL to downregulate HER2, which in turn prevented the proliferation of tumor cells (Li et al., 2007). The rare germline mutations in the CBL gene, which plays a role in the RAS-MAPK signaling pathway, have been linked to a condition known as neuro-heart-facial-skin syndrome. Helen L. Hanson documented the case of a girl who developed a Noonan-like phenotype and then mixed germ cell/teratoma in the ovary, which is followed by mature teratoma in the liver, retina, and ovary, all due to a novel heterozygous mutation in CBL. Notably, the loss of copy neutrality of these heterozygous CBL mutations occurred due to acquired haploidy at 11q23 in three of the teratomas, suggesting a specific association between CBL mutations and the susceptibility to germ cell tumors (Hanson et al., 2014).

NCK1 Divergent transcript (NCK1-DT or NCK1-AS1) is a recently identified long non-coding RNA (lncRNA) with the previously documented roles in promoting both tumor growth and resistance to cisplatin (DDP) in cervical cancer. Recent investigations have unveiled that NCK1-AS1 exerts its effects by stabilizing the NCK1 protein. It achieves this by directly interacting with c-CBL and inhibiting the c-CBL-induced degradation of NCK1. Interestingly, when NCK1 is upregulated, it can reverse the effects of inhibiting NCK1-AS1, thus affecting the biological behavior and DDP resistance of ovarian cancer cells. This groundbreaking study provides a promising way of developing novel treatments for ovarian cancer and overcoming chemoresistance (Chang et al., 2020).

### 3.12 Breast cancer

According to statistics, breast cancer accounts for 7%–10% of all malignant tumors, making it one of the most prevalent malignant tumors in females. CBL has an inhibitory effect on the proliferation and migration of breast cancer cells. According to an exploratory examination of the breast cancer patients with high RET expression, the high levels of CBL-c are linked to a favorable prognosis. According to these findings, CBL-c can ubiquitinate and downregulate RETMEN2A. Besides, it can mediate ubiquitination and degradation by inhibiting CBL (Kales et al., 2014). The tumor-inhibitory activity of transforming growth factor (TGF) can be dramatically inhibited by the breast cancer cells with high CBL expression. The expression of TGF- target genes, pai-1, and CDK inhibitors increased when CBL was downregulated. The loss of CBL reduces the ability of breast cancer cells to cause malignancy (Yarden, 2001; Kang et al., 2012).

The ubiquitination and regulatory processes that are exhibited by the CBL family and egfr are linked. It was discovered earlier that the egfr-induced apoptosis of MDA-MB-468 breast cancer cells was reduced by the overexpression of CBL-b. By preventing the proteasome from degrading EGFR, the inhibitory effects of CBL-b overexpression on apoptosis and EGFR signaling can be reversed (Ettenberg et al., 1999). Early research has demonstrated that HER-2/ErbB-2 overexpression is linked to a poor prognosis, and the capacity of HER2-specific antibodies to direct HER2 into CBL-dependent endocytosis and degradation pathways is related to their anti-tumor effects (Li et al., 2007). Tyrosine kinase transmembrane receptor ErbB2 overexpression is seen in a wide range of tumors, including breast and ovarian cancers. ErbB2 is resistant to the c-CBL-mediated degradation and c-CBL ligand-induced ubiquitination of ErbB1 (epidermal growth factor receptor) (Klapper et al., 2000).

Several studies have revealed a significant association between CDC42 and CBL-c. CDC42 is frequently overexpressed in many cases of breast cancer, and evidence suggests that activated CDC42 enhances the accumulation of ErbB1 within cells by modulating the function of c-CBL. Conversely, when c-CBL is inhibited, it leads to an increase in EGFR levels. CDC42 and c-CBL are the critical components involved in the regulation of EGFR protein levels, and restoring proper EGFR degradation by disrupting the regulation of c-CBL by CDC42 can effectively reduce the proliferation and migration of breast cancer cells (Hirsch et al., 2006). In another study, it was discovered that the REDOX/Fyn/c-CBL (RFC) pathway, which typically translates the slight increase in oxidation into the accelerated degradation of c-CBL target proteins, was inhibited by Cdc42 in basal-like breast cancer (BLBC) cells (Schmidt et al., 2006; Chen et al., 2013). The competition of Erb signal heterodimers for binding to c-CBL promotes the migration. Meanwhile, EphA2 is promoted by c-CBL to degrade in response to stimuli. These results lay a foundation for the further research of EphA2 as a possible target for therapeutic intervention (Walker-Daniels et al., 2002; Hirsch and Wu, 2007).

Breast, colon, lung, and pancreatic cancers show higher levels of CTEN mRNA, according to the analysis of public databases. By binding to the E3 ubiquitin ligase c-CBL and reducing EGFR ubiquitination, CTEN suppresses ligand-induced EGFR degradation (Hong et al., 2013). We looked at the expression and tyrosine phosphorylation of the c-CBL proto-oncogene product in several human cancer cell lines and surgical tissues for identifying the signaling pathways linked to human carcinogenesis. Of the 36 surgical specimens taken from human tumor tissue, the c-CBL protein was shown to be tyrosine phosphorylated in 12 (33%) cases. These findings demonstrate the significance of c-CBL signaling to the development of human tumors (Kamei et al., 2000). By activating c-CBL and ERK to downregulate EZH2, YC-1 causes the apoptosis of breast cancer cells and reduces tumor development (Chang et al., 2014). It was discovered by using a yeast double mixing sieve that the Cool home binding partner was CBL-b. The interactions between Cool-CBL-b require the SH3 domain of Cool and conflict with the binding of PAK to Cool proteins. In addition to providing an alternative mechanism, the expression of CBL-b also efficiently prevents Cool-2 from stimulating PAK, which in turn catalyzes the ubiquitination of the receptor. It was discovered that CBL-b can inhibit the signal activity that requires PAK activation (Flanders et al., 2003).

Breast cancer progression is significantly influenced by the RANKL/RANK pathway, which is a receptor activator of nuclear factor-B. CBL-b pathway is a crucial regulator of RANKL/RANK signaling. In the study of Yunzhang Ling, it was discovered that CBL-b negatively regulated the Src-Akt/ERK pathway and suppressed the expression of RANK protein in both human breast cancer tissue samples and animal samples. By preventing RANKL-induced breast cancer cell migration and metastasis, CBL-b enhances the prognosis of the breast cancer patients who express RANKL (Zhang L. et al., 2015). In MDR breast cancer and gastric cancer cells, CBL-b was poorly expressed. Additionally, in MDR cell cultures, the overexpression of CBL-b decreased cell migration both *in vitro* and *in vivo*. By ubiquitinating and degrading EGFR, the overexpression of CBL-b also inhibits the EGFR-ERK/Akt-miR-200c-ZEB1 axis, which in turn suppresses EMT (Xu et al., 2017a).

Drug resistance is still a significant issue in the conventional chemotherapy medicines used to treat breast cancer (Liu W. J. et al., 2023). Through a methodical approach, it was found out that the breast cancer cells that are responsive to broad-spectrum anticancer medications *in vitro* and *in vivo* play a significant role in microRNA-27b-3p (miR27b), which is absent from breast cancer tissues and cell lines. By specifically targeting CBL-b and GRB2 and inactivating the PI3K/Akt and MAPK/Erk signaling pathways, miR-27b can, however, improve the response to PTX. The research offers a prescription for dealing with the treatment resistance in breast cancer patients (Chen et al., 2018; Li W. et al., 2018). CBL is significant in the therapy of breast cancer because it engages HIF-1 for ubiquitination in a proteasoma-dependent manner (Xiong et al., 2022).

EphB6 expression not only promotes actin-dependent migration and adhesion but also inhibits the invasiveness of aggressive breast cancer cells. A new role of c-CBL in preventing tumor invasion has been discovered. It can silence and inhibit Abl phosphorylation, cell adhesion, and morphological change, in addition to limiting the capacity of EphB6 to suppress invasion (Truitt et al., 2010; Song et al., 2022). A more precise clinical prediction model for OS and DFS in breast cancer patients can be constructed through the expression of CBL-b (Liu et al., 2020). Compared to healthy tissues and cells, CBL-c was expressed at a higher rate in breast cancer tissues and cells. The patients with breast cancer have a better prognosis when CBL-c expression is higher. CBL-c has the ability to stop breast cancer cells from proliferation, migration, and invasion.

In IP tests, several researchers discovered that CBL-c and CTTN interacted in the cytoplasm. CTTN is degraded more rapidly by the ubiquitin-proteasome pathway due to CBL-c, although its mRNA level is unaffected. The inhibitory effect of CBL-c on the proliferation, migration, and invasion of breast cancer cells was partially reversed by CTTN. Also, the interaction with CIN85 recruits c-CBL to the AMAP1 complex. The invasive phenotypic development and stromal destruction of cancer cells depend on its ubiquitination activity (Yan et al., 2012; Sato et al., 2013). The findings imply that CIN85 and CBL, two well-known growth factor receptor signaling inhibitors, may be actively involved in tumor invasion and that the role of AMAP1 in breast cancer cell invasion is mediated by intricate epigenetic mechanisms (Yang et al., 1991). Furthermore, disrupting the interaction of CBL mutations with EGFR or CIN85 can also reduce the development of cancer. Breast cancer progression is synchronized with CIN85 by the high expression of inactivated CBL. To sum up, these findings provide a theoretical framework for investigating the possibility of EGFR-CBL-cIN85 axis targeting in CBL-inactivated mutant malignancies (Ahmed et al., 2021).

Because of its anti-estrogen effect, the non-steroidal anti-estrogen tamoxifen (TAM) is frequently used to treat breast cancer. MCF-7 cells underwent time-dependent apoptosis as a result of high TAM concentrations. This process results in an upregulation of ubiquitin ligase c-CBL. While 70Z/CBL prevented the apoptosis induction of TAM, and the overexpression of c-CBL dramatically increased the induction of apoptosis in TAM. Additional research revealed that c-CBL overexpression dramatically reduced c-Src protein levels and TAM-induced AKT activation. According to studies, c-CBL regulates c-Src expression and TAM-induced ERK and AKT activity to make MCF-7 cells more sensitive to TAM (Yan et al., 2011).

In clinical practice, the precursor medication hydroxyanilide (SAHA) is now utilized to treat cancer. Through the ubiquitin-proteasome pathway, SAHA promotes the destruction of the Tie2 protein. After SAHA treatment, the expression of the E3 ligase c-CBL, which is required for Tie2 ubiquitination, increases rapidly. The tie2 protein degradation caused by SAHA was stopped by c-CBL knockdown (Zhu et al., 2020). By enhancing c-CBL and chip-mediated HER2 ubiquitination and subsequent proteasome or lysosomal HER2 degradation, acetyltanshinone IIA (ATA) promotes HER2 destruction. c-CBL can reverse tamoxifen resistance in HER2-overexpressed breast cancer cells by inhibiting the formation of ER-c-Src-HER2 complex (Li W. et al., 2018; Huang et al., 2020). By controlling the H19-miR-675-5p-CBL axis, hurir extract reduced the viability of breast cancer cells and triggered apoptosis (Melani et al., 1989).

## 4 Potential treatment for CBL family drugs

CBL proteins also play an important role in anti-tumor targeted drugs and many natural products. In recent years, the development of anti-tumor drugs based on CBL family proteins has become a focus of attention, and many related drugs are currently under research or preclinical research, including western drugs, but also include natural product drugs (Table 2).

**TABLE 2:** Potential therapeutic approaches.

Drug		Types	Signaling pathway	References
Western medicine	DEX	CBL	gsk-3β	Jeon et al. (2020)
	Licochalcone A	c-CBL	Met	Han et al. (2022)
	Dasatinib	CBL	EGFR	Chang et al. (2008)
	Cetuximab	CBL-b	EGFR	Yu et al. (2016)
	5-fluorouracil	CBL-b	EGFR, ERK, Akt	Feng et al. (2013)
	Olaparib	CBL-c	EGFR	Frankum et al. (2015)
	Flavones	c-CBL	Notch	Zhu et al. (2020)
Natural product	Bufalin	CBL-b	Akt/mTOR/p70S6K	Qi et al. (2019)
	β-elemene	c-CBL, CBL-b	Akt	Zhang et al. (2013b)
	Proanthocyanidins	c-CBL	EGFR	Daveri et al. (2021)
	Berberine	c-CBL	β-catenin	Ruan et al. (2017)
	Chloroquine	c-CBL	TRAIL	Park et al. (2016)
	Acety tanshinone IIA	c-CBL	EGFR	Huang et al. (2020)
	Arsenic trioxide	CBL-b	PI3K/Akt	Zhang et al. (2012)

### 4.1 Western medicine

Prior research results showed that DEX mediated the upregulation of CBL through gsk-3ß pathway, thereby reducing the protein stability of DR5. Ultimately, the apoptosis of human lung cancer cells (A549) was induced (Jeon et al., 2020). Licochalcone A (Lico A), a natural product, has demonstrated significant inhibitory effects against a wide range of tumors. Lico A facilitates the interaction between c-Met and CBL, leading to the augmentation of c-CBL-mediated ubiquitination and the degradation of c-Met. Notably, the depletion of c-CBL compromises the ability of Lico A to induce c-Met ubiquitination and diminishes its inhibitory effect on gefitinib-resistant non-small cell lung cancer cells (Han et al., 2022).

Recent studies have highlighted the potent activity of dasatinib, a multi-target kinase inhibitor, against EphA2. Dasatinib effectively inhibits the binding of ligands to EphA2 and prevents its interaction with the ubiquitin ligase CBL. Additionally, dasatinib hinders the internalization and degradation of EphA2. These findings suggest that dasatinib plays a crucial role in pancreatic cancer by acting as an inhibitor of EphA2 (Chang et al., 2008). Cetuximab is a monoclonal antibody targeting the epidermal growth factor receptor (EGFR). In the context of gastric cancer, the involvement of CBL-b in the apoptosis of gastric cancer cell lines MGC803 and BGC823 as induced by Cetuximab has been observed. Silencing CBL-b expression specifically leads to an increase in EGFR expression. These findings indicate that CBL-b plays a role in modulating the sensitivity of gastric cancer cells to cetuximab. In other words, targeting CBL-b can enhance the effectiveness of cetuximab in treating gastric cancer (Yu et al., 2016).

5- fluorouracil (5-FU) is a crucial chemotherapy agent used to treat gastric cancer. Within this context, the ubiquitin ligase CBL-b serves as a negative regulator of growth factor receptor signaling pathways, thus impeding the proliferation of cancer cells. Research indicates that reducing the expression of CBL-b can promote cell proliferation and hinder cell apoptosis in the gastric cancer cells treated with 5-FU. As revealed by mechanistic investigations, the knockdown of CBL-b leads to a significant increase in the phosphorylation of EGFR, ERK, and Akt, along with a reduction in mitochondrial membrane potential and an increase in Bcl-2/Bax expression. These findings collectively suggest that CBL-b augments the sensitivity of gastric cancer cells to 5-FU through EGFR and mitochondria-mediated pathways (Feng et al., 2013).

Recently, the PARP1 inhibitor olaparib received approval for the treatment of ovarian cancer patients with BRCA1 or BRCA2 mutations. Jessica Frankum made an important discovery in this regard, identifying the E3 ubiquitin ligase CBL-c as a potential biomarker for predicting the response to olaparib. This discovery was made through the use of two parallel RNA interference screening methods. This finding highlights the significance of CBL-c to determining the efficacy of olaparib and its potential as a biomarker in selecting patients for ovarian cancer treatment (Frankum et al., 2015). Flavones have been found to upregulate the levels of c-CBL, a protein involved in cellular signaling. This up-regulation enhances the interaction between c-CBL and ICN1, a key protein in Notch signaling pathway. Consequently, ICN1 undergoes ubiquitination and degradation. In the context of T-cell acute lymphoblastic leukemia (T-ALL), the downregulation of ICN1 induced by flavonoids can be reversed by the knockdown of c-CBL. Moreover, the inhibited proliferation of T-ALL cells is observed when c-CBL is suppressed. Therefore, the modulation of c-CBL levels by flavones presents a potential therapeutic strategy to counter T-ALL by regulating ICN1 and impeding cell proliferation (Zhu et al., 2020).

### 4.2 Natural product

Bufalin, a compound naturally derived from traditional Chinese medicine, exhibits potent anti-cancer effects on various cancer cells through different mechanisms. Specifically, bufalin induces autophagy in MGC803 cells by modulating the Akt/mTOR/p70S6K and ERK signaling pathways. In this process, CBL-b plays a positive regulatory role by inhibiting mTOR activity and promoting the activation of ERK1/2. Overall, these findings demonstrate that bufalin induces autophagy in MGC803 cells through multiple signaling pathways, with CBL-b as a key regulator of autophagy progression (Qi et al., 2019). Additionally, bufalin is known to upregulate the ubiquitin ligase family of CBL, which acts as an upstream regulator of PI3K. This regulatory effect on CBL provides further insights into the mechanism by which bufalin modulates the PI3K/Akt pathway to affect the cellular processes associated with this signaling cascade (Li D. et al., 2009).

Recent research has revealed that β-elemene, a compound derived from traditional Chinese medicine, possesses anti-tumor properties, particularly in overcoming multidrug resistance. Additionally, the expression of c-CBL and CBL-b, the members of the E3 ubiquitin ligase family, is significantly upregulated immediately after β-elemene treatment. These findings suggest that β-elemene can affect cellular signaling pathways, thus enhancing the ubiquitination and degradation of target proteins potentially through the modulation of c-CBL and CBL-b expression. This mechanism may contribute to the ability of β-elemene to counteract multidrug resistance in cancer cells (Zhang Y. et al., 2013).

Arsenic trioxide (ATO) exhibits different molecular mechanisms in the treatment of hematological malignancies and certain solid tumors. Its therapeutic effects are attributed to a wide range of biological processes and pathways. FLIP acts as a suppressor of apoptosis that functions through death receptors. The study revealed a novel link between the FLIP(L) downregulation of cells and ATO-induced autophagy. ATO induces FLIP(L) degradation in K562 and MGC803 cells through the ubiquitin-proteasome pathway. In addition, CBL-b is involved in this process and interacts with FLIP(L) to promote the proteasome degradation of FLIP(L) (Zhang et al., 2012).

Gastric cancer cells exhibited a reduction in the sensitivity to ATO compared to other cell types. ATO treatment led to an increase in CBL protein expression in both the NB4 and MGC803 cell lines. Notably, when CBL was inhibited using the proteasome inhibitor Ps341, the apoptosis of NB4 cells decreased, while the G2/M phase arrest of MGC803 cells increased. Moreover, inhibiting CBL prolonged the activation of the PI3K/Akt pathway induced by ATO. These findings suggest that CBL plays a crucial role in mediating the effects of ATO. By inhibiting the PI3K/Akt signaling pathway, CBL is involved in the ATO-induced apoptosis in NB4 cells and G2/M phase arrest in gastric cancer cells. The results further highlight the significance of the CBL-mediated regulation of PI3K/Akt signaling to the cellular responses to ATO treatment (Li Y. et al., 2009).

The consumption of dietary proanthocyanidins (PAC) is linked to a lower risk of developing colorectal cancer (CRC). The epidermal growth factor receptor (EGFR) signaling pathway is frequently disrupted in various types of cancers, including colorectal cancer, thus leading to its dysregulation. Hexameric PAC (Hex) exhibits anti-proliferative and pro-apoptotic properties in human colorectal cancer cells. This phosphorylation at Tyr 1045 creates a binding site for the ubiquitin ligase c-CBL, facilitating the degradation of EGFR within lysosomes. Consequently, this degradation hampers the growth of colorectal cancer (CRC) cells (Daveri et al., 2021). Berberine, an isoquinoline alkaloid, has long been used in traditional Chinese medicine for its efficacy in alleviating the symptoms of diarrhea and gastroenteritis. This effect is achieved through the downregulation of the β-catenin signaling pathway. Berberine binds to specific receptors, facilitating the interaction between retinoid X receptor-alpha (RXRα) and nuclear β-catenin. Consequently, this interaction triggers the degradation of β-catenin mediated by c-CBL, thus inhibiting colon cancer cell proliferation (Ruan et al., 2017).

Chloroquine (CQ) is a medication commonly used for the treatment of malaria. In recent studies, it has been observed that CQ has the ability to reduce the expression of the E3 ligase CBL, which is associated with the death receptor 5 (DR5). The decrease in CBL levels leads to a significant increase in the upregulation of DR5. Furthermore, the inhibitory effects of CQ on lysosomes result in the upregulation of DR5-mediated TRAIL (tumor necrosis factor-related apoptosis-inducing ligand)-mediated apoptosis in human renal carcinoma Caki cells (Park et al., 2016). Research has demonstrated that acetyl tanshinone IIA (ATA) facilitates the degradation of HER2, a protein associated with cancer, through various mechanisms. ATA has been shown to enhance the activity of c-CBL and CHIP, two enzymes involved in the ubiquitination of HER2. This increased ubiquitination leads to the subsequent degradation of HER2, either through the proteasome pathway or the lysosomal pathway (Huang et al., 2020).

## 5 Immunotherapy

Immunotherapy refers to the treatment of low or high immune state of the body, artificially enhance or suppress the immune function of the body to achieve the purpose of treating diseases. There are many methods of immunotherapy, which are suitable for the treatment of many diseases. Tumor immunotherapy aims to activate the body’s immune system and kill cancer cells and tumor tissue by relying on its own immune function. Immunotherapy has revolutionized the field of cancer treatment. While most immunotherapies today are antibodies that target membrane checkpoint molecules, there is an increasing need for small molecule drugs that address intracellular pathways (Hu et al., 2024). CBL-b is expressed in several immune cell lineages, and it negatively regulates the activity of immune cells. CBL-b has been specifically identified as an attractive target for cancer immunotherapy because of its role in promoting an immunosuppressive tumor environment.

CBL-b regulates downstream proteins of multimembrane receptors and coreceptors, limiting activation of the innate and adaptive immune system (Zhou et al., 2024). CBL-b is also the downstream master regulator for CD28 and CTLA-4 signals. This E3 ubiquitin ligase regulates innate and adaptive immune cells, ultimately promoting the immunosuppressor tumor microenvironment (TME) in the absence of CD28 co-stimulation (Kimani et al., 2023). CBL-b plays an important role in setting appropriate thresholds for activation of T cells and controlling tolerance of peripheral T cells through various mechanisms. Overexpression of CBL-b results in a low immune response from T cells. Conversely, the lack of CBL-b in T cells led to a significant increase in IL-2 production, even in the absence of CD28 co-stimulation *in vitro* (Augustin et al., 2023). In summary, CBL-b may be involved in immune-mediated diseases, and blocking CBL-b can be considered as a novel anti-tumor immunotherapy strategy. New CBL-b inhibitors provide antigen-specific immune stimulation and are promising therapeutic tools in the field of immunooncology.

## 6 Conclusion

The ubiquitination-proteasome system represents a fundamental cellular process and has attracted much attention as a potential drug target. Many preclinical and clinical trials are indeed underway to test compounds for a variety of diseases. In this case, CBL is an ideal candidate, and this paper comprehensively reviews the role of the CBL family in tumorigenesis and development.

The CBL family is a group of E3 ubiquitin ligases, which has an important relationship with the occurrence and development of tumors. The CBL family plays a crucial role in regulating signaling pathways associated with tumor development. The CBL family influences key cellular processes in tumor growth and metastasis by controlling the degradation and signaling of RTKs. These insights highlight the importance of the CBL family in complex signal regulatory networks and its potential as intervention targets for cancer therapy, but the underlying molecular mechanisms by which the CBL family regulates RTK still require further investigation.

In recent years, CBL has also played an important role in anti-tumor targeted drugs and many natural products, and there are still many related drugs under research or preclinical research. In addition, the important role of cbl family proteins in immunotherapy has been widely demonstrated, and the anticancer effects of CBL proteins can be targeted by specifically modifying their ability to bind to various cancer-causing proteins, making them attractive targets for cancer therapy. However, little is known about the regulatory mechanism of CBL in human immune cells. Some unexplored aspects of CBL biology, such as biomarkers of its activity and its role in other cancer-generating models, still need further study. These may provide new clues for future clinical applications, and provide new ideas and breakthroughs for tumor treatment targets.
